# Reduction of NADPH-Oxidase Activity Ameliorates the Cardiovascular Phenotype in a Mouse Model of Williams-Beuren Syndrome

**DOI:** 10.1371/journal.pgen.1002458

**Published:** 2012-02-02

**Authors:** Victoria Campuzano, Maria Segura-Puimedon, Verena Terrado, Carolina Sánchez-Rodríguez, Mathilde Coustets, Mauricio Menacho-Márquez, Julián Nevado, Xosé R. Bustelo, Uta Francke, Luis A. Pérez-Jurado

**Affiliations:** 1 Departament de Ciències Experimentals i de la Salut, Universitat Pompeu Fabra, Barcelona, Spain; 2 Centro de Investigación Biomédica en Red de Enfermedades Raras (CIBERER), ISCIII, Madrid, Spain; 3 Unidad de Investigación, Hospital Universitario de Getafe, Getafe, Spain; 4 Centro de Investigación del Cáncer e Instituto de Biología Molecular del Cáncer de Salamanca (IMBCC), CSIC–Universidad de Salamanca, Salamanca, Spain; 5 INGEMM-Instituto de Genética Médica y Molecular/IdiPAZ, Madrid, Spain; 6 Department of Genetics, Stanford University School of Medicine, Stanford, California, United States of America; University of Oxford, United Kingdom

## Abstract

A hallmark feature of Williams-Beuren Syndrome (WBS) is a generalized arteriopathy due to elastin deficiency, presenting as stenoses of medium and large arteries and leading to hypertension and other cardiovascular complications. Deletion of a functional *NCF1* gene copy has been shown to protect a proportion of WBS patients against hypertension, likely through reduced NADPH-oxidase (NOX)–mediated oxidative stress. DD mice, carrying a 0.67 Mb heterozygous deletion including the *Eln* gene, presented with a generalized arteriopathy, hypertension, and cardiac hypertrophy, associated with elevated angiotensin II (angII), oxidative stress parameters, and *Ncf1* expression. Genetic (by crossing with *Ncf1* mutant) and/or pharmacological (with ang II type 1 receptor blocker, losartan, or NOX inhibitor apocynin) reduction of NOX activity controlled hormonal and biochemical parameters in DD mice, resulting in normalized blood pressure and improved cardiovascular histology. We provide strong evidence for implication of the redox system in the pathophysiology of the cardiovascular disease in a mouse model of WBS. The phenotype of these mice can be ameliorated by either genetic or pharmacological intervention reducing NOX activity, likely through reduced angII–mediated oxidative stress. Therefore, anti-NOX therapy merits evaluation to prevent the potentially serious cardiovascular complications of WBS, as well as in other cardiovascular disorders mediated by similar pathogenic mechanism.

## Author Summary

Williams-Beuren Syndrome (WBS) is a rare developmental disorder characterized by distinctive facial, neurobehavioral, and cardiovascular features, caused by a heterozygous loss of genetic material (deletion) at the 7q11.23 chromosomal band. Elastin protein deficiency, due to deletion of one copy of the *ELN* gene, is responsible for developmental anomalies in arterial wall remodeling, predisposing WBS patients to high blood pressure and other serious cardiovascular complications. We have previously shown that a fraction of WBS patients who lack a copy of the *NCF1* gene, which codes for p47*^phox^*, a subunit of NADPH-oxidase (NOX), have lower cardiovascular risk associated with decreased oxidative stress. Here, we used a mouse model of elastin deficiency to better define the contribution of NOX–mediated oxidative stress to the cardiovascular phenotype of WBS and to confirm the role of *Ncf1* as a major modulator. In addition, pharmacological inhibition of NOX activation or synthesis with either losartan or apocynin significantly rescued the cardiovascular phenotype of these mice, suggesting that these drugs should also be evaluated in human patients.

## Introduction

Williams-Beuren syndrome (WBS [MIM 194050]) is a developmental disorder with multisystemic manifestations and a prevalence of ∼1/10,000 newborns, caused by a segmental aneusomy of 1.55–1.83 Mb at chromosomal band 7q11.23, which includes *ELN* (coding for *elastin* [MIM 130160]) and 25–27 additional genes [1], [2]. The recurrent WBS deletion common to most patients is mediated by nonallelic homologous recombination between regional segmental duplications that flank the WBS critical region [3]. In addition to distinctive craniofacial characteristics and mild mental retardation with social disinhibition and hyperacusis, a hallmark feature of WBS is a generalized arteriopathy presenting as narrowing of the large elastic arteries [4]. Histological characterization of arterial vessel walls of WBS patients showed increased number and disorganized lamellar structures, fragmented elastic fibers, and hypertrophy of smooth muscle cells [5]. This large arterial vessel remodeling which is a consequence of abnormalities in vascular development, is thought to be responsible for the cardiovascular disease manifested in 84% of WBS patients [4], [6]. Identical vascular features, most prominently supravalvular aortic stenosis, are also found in patients with heterozygous deletions or disruptions of the *ELN* gene, implicating elastin haploinsufficiency in this phenotype [5], [7]. The arteriopathy is the main cause of serious morbidity in WBS, including systemic hypertension and possible complications such as stroke, cardiac ischemia, and sudden death [8], [9].

Animal models provide further evidence for elastin deficiency as the main cause of cardiovascular disease in WBS, underscoring the prominent role of the elastic matrix in the morphogenesis and homeostasis of the vessel wall [10]. Heterozygous knockout mice with only one copy of the *Eln* gene reproduce many of the alterations observed in the WBS vascular phenotype [11], [12]. Hypertension is a consistent feature of *Eln* ^+/−^ mice, associated with elevated plasma renin activity (PRA) and angiotensin II (angII) levels, that can be blocked by the administration of angII type 1 receptor (AT1R) antagonists [13]. In addition to direct effects on the vasculature, many of the cellular actions of angII are mediated by the activation of the NADPH-oxidase (NOX), thus stimulating the formation of reactive oxygen species (ROS). Evidence is accumulating that increased oxidative stress has a relevant pathophysiological role in cardiovascular disease, including hypertension, atherosclerosis, and heart failure [14].

In WBS, the dosage of the *NCF1* gene, encoding the p47^phox^ subunit of NOX, is a strong modifier of the risk of hypertension. Hypertension was significantly less prevalent in patients whose deletion included *NCF1*, indicating that hemizygosity for *NCF1* was a protective factor against hypertension in WBS. Decreased p47^phox^ protein, superoxide anion production, and protein nitrosylation levels, were all observed in cell lines from patients hemizygous at *NCF1* [15]. Reduced angII-mediated oxidative stress in the vasculature was the proposed mechanism behind this protective effect. Indeed, studies performed in *Ncf1* knockout mice have revealed that p47^phox^ is one of the major effectors of angII action. The administration of angII did not lead to increased superoxide production or blood pressure elevation in homozygous knockout animals, as it did in wild-type mice [16].

The aim of the present study was to evaluate whether oxidative stress significantly contributes to the cardiovascular phenotype of a mouse model for WBS, and whether reduction of NOX activity by genetic modification and/or by pharmacological inhibition might have a potential benefit in the rescue of this phenotype. By using non-invasive blood pressure measurements, histological, biochemical and molecular analyses, we have documented a negative correlation between NOX activity and the cardiovascular phenotype in a mouse model of WBS, as well as prevention of many of the manifestations by using anti-NOX therapies.

## Results

### Cardiovascular phenotype of DD mice related to elevated angII and oxidative stress

Previously reported mice bearing a heterozygous deletion of half of the orthologous region of the WBS locus (0.67 Mb, from *Limk1* to *Trim50*, including *Eln*), called DD, were used as a model for the WBS cardiovascular phenotype [11], [17].

We confirmed the elevated systolic, diastolic and mean blood pressures of 16-weeks old DD mice, ∼40% higher than their wild-type littermates on average, without increased heart rate (Table 1 and Table S1). Hypertension was already present at 8 weeks of age and persisted throughout life (Table S2) without reducing life-expectancy, since these animals have been kept alive for more than 2 years with no instances of early death or unexpected morbidity [11]. As previously reported [17], body weight was significantly reduced for DD mice at all ages when compared to wild-type (*P*<0.001) (Table 1).

**Table 1:** Cardiovascular parameters of 16-week-old wild-type and DD mutant mice.

Parameter	Wild-type	DD
Systolic BP (mmHg)	114.58±7.68	151.43±12.67**
Diastolic BP(mmHg)	87.80±8.89	129.53±10.08**
Mean BP (mmHg)	96.18±8.08	135.94±10.78**
Heart rate (bpm)	613±23.92	625.6±31.28 (NS)
Body weight (g)	33.59±0.86	27.71±0.63**
Heart weight (g)	0.19±0.01	0.24±0.01*
%Heart weight / Body weight	0.56±0.11	0.89±0.11**
LV Cardiomyocyte size (µm^2^)	240.70±17.70	313.52±2.12**
RV Cardiomyocyte size (µm^2^)	322.08±18.04	433.29±20.24**
Aorta wall thickness (µm)	61.61±6.98	77.61±5.70**
Lamellar units in aorta	7.12±0.7	8.39±1.38*
AngII levels (pg/ml plasma)	48.27±6.67	82.24±7.39**
Protein Nitrosylation (a.u.)	1.6×10^4^±3.3×10^3^	2.3×10^4^±1.1×10^3^ **
Superoxide anion (a.u.)	1.2×10^4^±4.2×10^3^	1.7×10^4^±5.5×10^3^ *

NS: not significant change. BP, arterial blood pressure. a.u., Arbitrary Units.

*P<0.05;

**P<0.001 versus wild-type.

Post-mortem evaluation at 16 and 32 weeks revealed significantly larger hearts in DD mice, measured as the heart wet-weight relative to the body weight (*P*<0.001). The cardiac hypertrophy was associated with increased cardiomyocyte size both in left and right ventricles (*P*<0.001) (Table 1, Tables S3 and S4). Vascular histology and morphologic examination provided insight into the structure of the aorta. DD mice showed fragmented, disorganized and jagged elastin sheets when compared to wild-type vessels in sections of the ascending aortic wall stained with elastic VVG, as previously reported [11]. We also observed a significantly increased arterial wall thickness (*P*<0.001), with small changes in the number of lamellar units (Table 1).

The expression of three genes encoding components of the angII biosynthetic pathway, angII precursor (angiotensinogen, *Agt*), renin (*Ren*) and angII converting enzyme (*Ace*), was increased 2 to 5 fold by qRT-PCR on mRNA of several tissues (heart, aorta, lung and kidney) (Figure S1). Accordingly, DD mice showed significantly elevated angII peptide plasma levels (*P*<0.001) (Table 1). Plasma renin activity levels were, however, highly variable even within groups, thus preventing inter-group comparisons.

The level of oxidative stress in ascending aortas was determined using two experimental avenues, quantifying the levels of superoxide anion and protein nitrosylation (Table 1). These assays demonstrated higher levels of oxidative stress in DD mice when compared to those in wild-type littermates. These results confirm the relationship between hypertension, elevated angII and increased oxidative stress in these mice.

### *Ncf1* gene expression as a modulator of the cardiovascular phenotype

**Figure 1:**
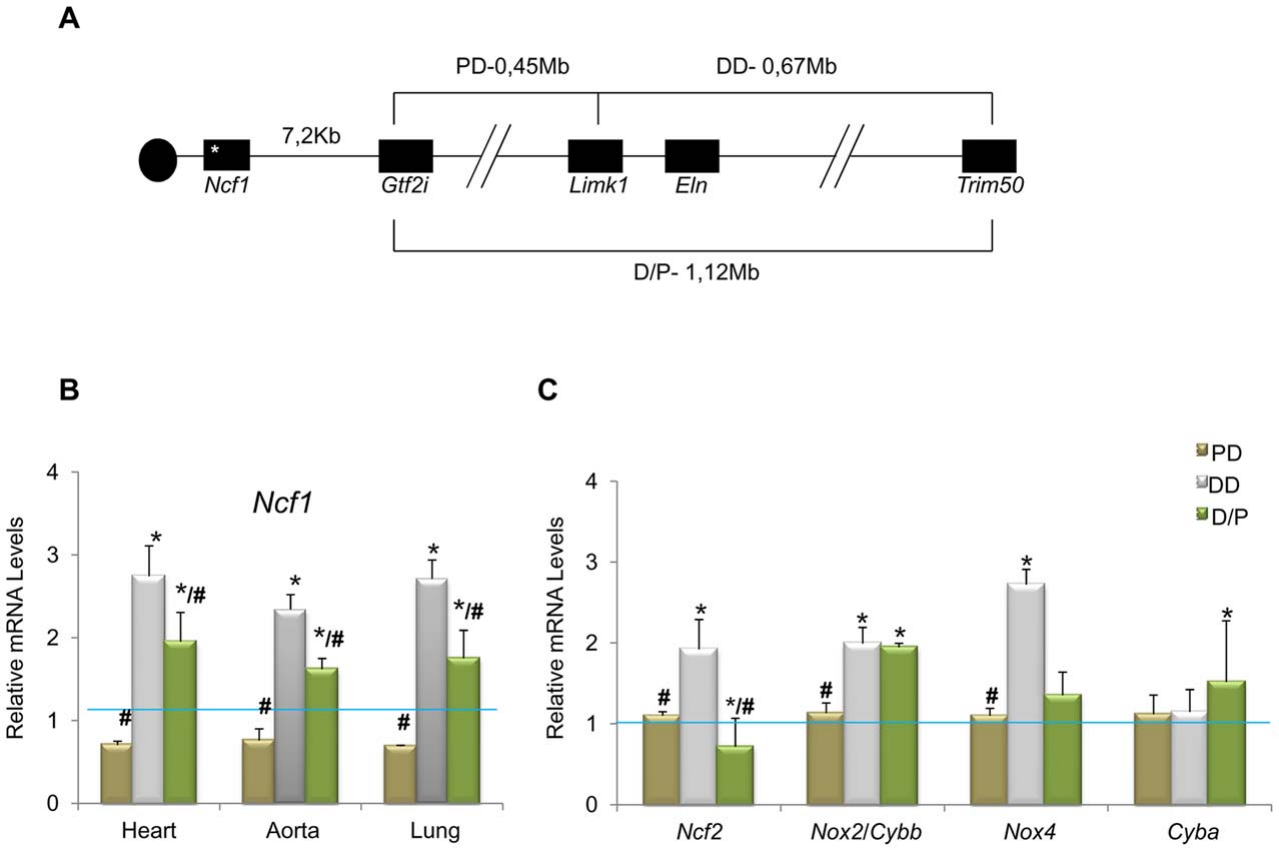
Genotypes of the different mouse models and relative transcript levels of oxidative stress genes. (A) Schematic map of the genomic structure of the orthologous region to the WBS locus in mouse and the rearrangements present in the different mouse models used in this study. (B) *Ncf1* transcript levels were significantly increased (∼3-fold) in DD animals compared to wild-type mice. *Ncf1* was down-regulated in PD animals, and over-expressed (∼2-fold) in P/D mice but significantly down-regulated with respect to DD mice. These differences were reproducible in three tissues tested; heart, aorta and lung. (C) Transcript levels of other oxidative stress genes in heart tissue of P0 mice. *Ncf2*, and *Nox4* transcripts were also significantly increased only in DD compared to wild-type mice, while *Cybb/Nox2* were significantly increased in both DD *and* P/D mice. Data were normalized to the mean of the wild-type group, set as 1.0 as indicated by the blue line. The results represent the mean ± SD of three independent experiments conducted in triplicate. Similar results were also obtained using aorta and lung mRNA (Figure S2). * *P*<0.05 *versus* wild-type; # *P*<0.05 *versus* DD.

*NCF1* gene dosage had been shown to modify the risk of hypertension in WBS patients [15]. Interestingly, while DD mice were consistently hypertensive, PD mice (heterozygous 0.45 Mb deletion, from *Gtf2i* to *Limk1*) were normotensive and mean blood pressure in D/P mice (harboring both deletions *in trans*) was only slightly increased by ∼10% [11]. Although the *Ncf1* gene is located outside the PD deletion (Figure 1A), we investigated whether the expression levels of *Ncf1* could be affected in these mice, by qRT-PCR in three different tissues and using *Eln* (hemizygously deleted in DD and D/P and not deleted in PD) as control. DD mice showed a ∼3 fold increase of *Ncf1* mRNA, while the expression was reduced in PD animals, and elevated but only ∼2 fold in D/P mice, correlating with the blood pressure (Figure 1B). The low basal *Ncf1* expression in PD and the relatively lower (compared to DD) in D/P strongly suggest that there may be a *cis* regulator element controlling *Ncf1* expression in the PD deletion. It also indicates that *Ncf1* is a strong modifier for the cardiovascular phenotype secondary to elastin deficiency.

We then investigated the expression of other genes related to the NOX system. On average, transcript levels of all genes but *Cyba* were significantly increased in DD animals with respect to wild-type. In contrast, they were not significantly different in PD mice, and D/P mice showed elevated expression of *Nox2* along with the ∼2-fold increase of *Ncf1* levels (Figure 1C and Figure S2). The elevated transcriptional NOX levels observed in DD mice could be the basis for the excessive ROS and protein nitrosylation documented in the aortic wall.

### The cardiovascular phenotype of DD mice is partially rescued upon reduction of *Ncf1* gene dosage

**Figure 2:**
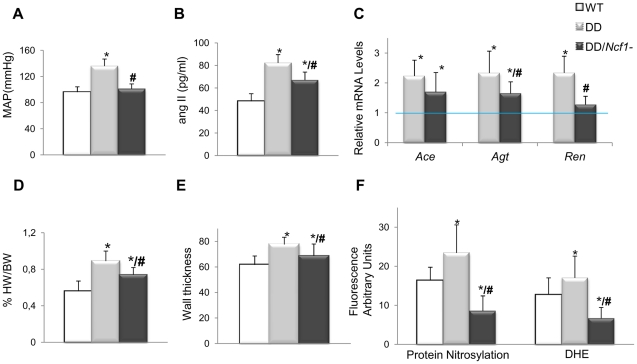
Cardiovascular features, angII/Ren system, and oxidative stress parameters in wild-type, DD, and DD/*Ncf1−* mice. (A) Mean arterial blood pressure in conscious 16-week-old mice. DD mice showed significantly elevated mean blood pressure when compared to wild-type littermates (*P*<0.001). Values were reduced in DD/*Ncf1−* animals with respect to DD, and were not significantly different from wild-type littermates. (B) AngII plasma levels were also significantly elevated in DD mice when compared to wild-type littermates (*P*<0.001) and partially reduced in DD/*Ncf1−* animals (*P* = 0.002/*P* = 0.036, versus wild-type and DD mice respectively). (C) Relative *Ace*, *Agt* and *Ren* transcript levels in heart mRNA from each genotype. Data were normalized such that the mean of the wild-type group was 1.0 represented by the blue line. (D) Relative heart weight with respect to body weight in post-mortem 16-week-old mice. DD mice displayed significantly enlarged hearts with respect to wild-type mice (*P*<0.001). Relative heart weight was ∼20% less in DD/*Ncf1−* compared to DD (*P* = 0.039). (E) Average arterial wall size in the ascending aorta of the different groups of animals. Arterial wall thickness was significantly smaller in DD/*Ncf1−* when compared to DD mice. (F) Quantification of oxidative stress parameters expressed as arbitrary unit of fluorescence. Significantly higher protein nitrosylation levels (*P*<0.001) and global ROS production (*P*<0.001) were found in DD mice compared with wild-type and DD/*Ncf1−* littermates. *P<0.05 *versus* wild-type; **^#^** *P*<0.05 *versus* DD mice. The results represent the mean ± SD (n = 7–12 per group or n = 3–4 per oxidative stress analysis).

We then crossed mice homozygous for a spontaneous loss of function mutation in *Ncf1* [18] with DD mice, in order to generate double heterozygotes *in trans* (DD*/Ncf1*−), resembling the genotype of WBS patients with lower risk of hypertension and deletions that include the *NCF1* locus. At 16 weeks of age, DD/*Ncf1*− animals had normal blood pressure similar to wild-type littermates (Figure 2A). AngII plasma levels were reduced with respect to DD mice (*P* = 0.036), although still elevated compared to wild-type values (*P* = 0.002) (Figure 2B), and were accompanied by a significant reduction of mRNA expression of the angII biosynthetic pathway genes (Figure 2C).

The hearts of DD/*Ncf1−* mice were 20% smaller than those of DD mice (*P* = 0.039), although they still were slightly larger than those of wild-type animals (*P* = 0.046) (Figure 2D and Table S3). Heart size reduction in DD/*Ncf1−* mice was accompanied with a decrease in the size of the cardiomyocytes of the left and right ventricles (*P* = 0.025 and 0.039 respectively). We also observed a reduction of the aortic wall thickness (*P* = 0.007), with a slight improvement in the organization of the elastin sheets (Figure 2E).

Expression of NOX-related genes was down regulated in DD*/Ncf1−* as compared to DD animals, reaching values similar to wild-type littermates. Consistently with these data, DD*/Ncf1−* mice showed significantly reduced levels of protein nitrosylation (*P*<0.001) and superoxide anion (*P*<0.001) in their ascending aortas compared to DD mice (Figure 2F).

### Pharmacological rescue of the cardiovascular phenotype of DD mice

**Figure 3:**
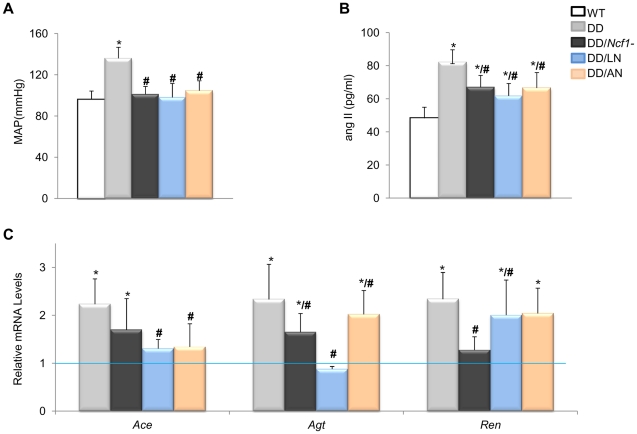
Effects of Losartan and Apocynin on blood pressure and the angII/Ren pathway. (A) Pre- and postnatal treatment with both losartan and apocynin effectively rescued the mean arterial blood pressure in conscious 16-week-old DD mice. (B) Treated DD mice had significantly reduced angII levels when compared to untreated DD littermates. (C) Relative *Agt*, *Ace* and *Ren* gene transcript levels in heart mRNA were also reduced in the losartan treated group when compared with untreated DD mice. Apocynin also showed moderate but significant effects. Data were normalized such that the mean of the wild-type group was 1.0 represented by the blue line. Only results from the post-natal onset groups for both treatments are represented, since data in the prenatal onset groups were very similar. *P<0.05 *versus* wild-type.; **#** *P*<0.05 *versus* untreated DD mice. The results represent the mean ± SD (n = 5–7 per group).

**Figure 4:**
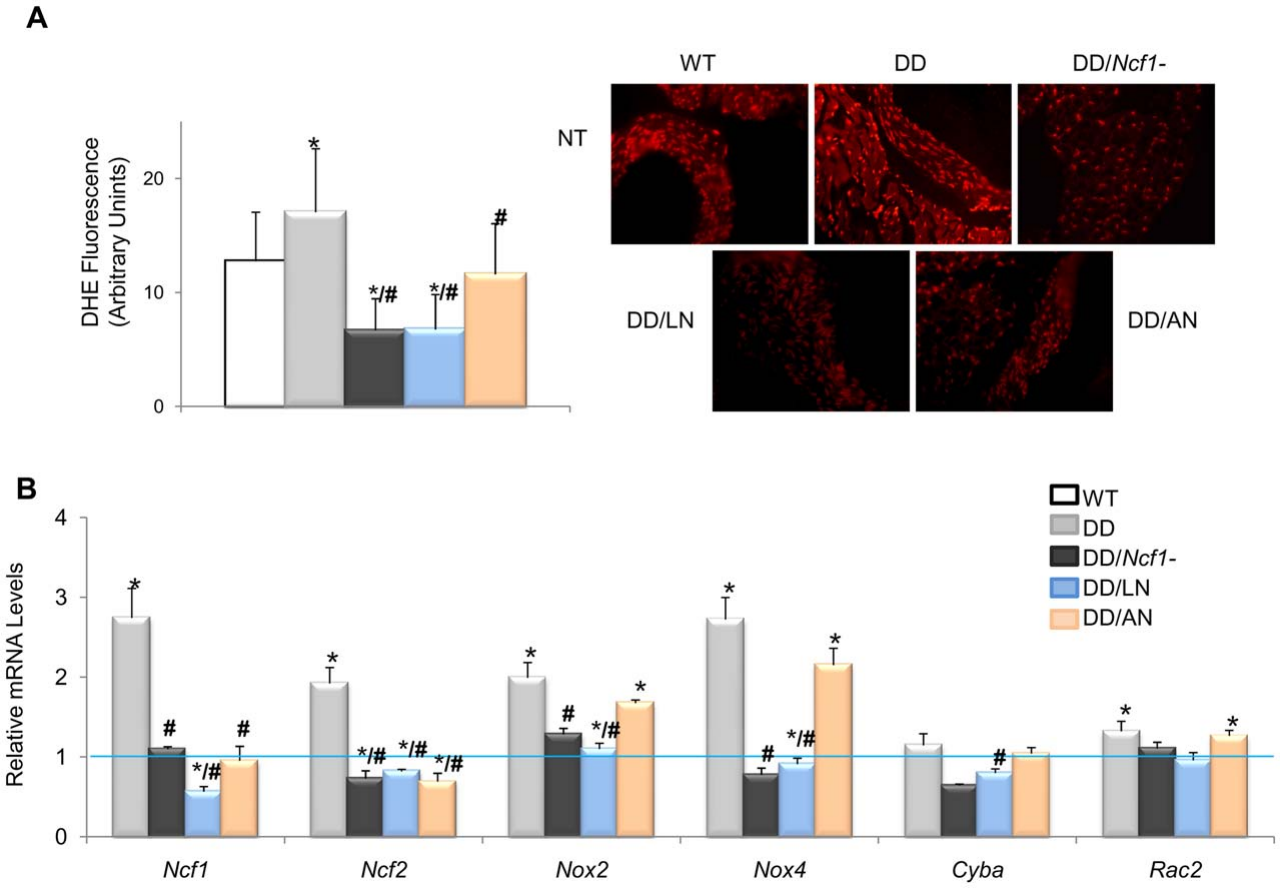
Oxidative stress parameters in ascending aortas of treated and untreated DD mice. (A) Quantification of DHE fluorescence intensity, expressed as arbitrary units of fluorescence. On the right, qualitative representation of superoxide levels in aortic sections stained with DHE. All images were analyzed using an Olympus BX-51 epifluorescence Microscope. Significantly lower global ROS (*P*<0.001) was found in treated mice. The results represent the mean ± SD of at least 20 measures taken in n = 3–4 animals per group. (B) Relative mRNA levels of NADPH-related genes. *Ncf1*, *Ncf2*, *Cyba* and *Nox4* transcripts were significantly lower in the treated animals with losartan or apocynin. Data were normalized such that the mean of the wild-type group was 1.0 represented by the blue line. Only results from the post-natal onset groups for both treatments are represented. *P<0.05 *versus* wild-type; #*P*<0.05 versus untreated DD mice.

The evidence for genetic complementation prompted us to investigate whether the treatment of DD mice with either losartan (an AT1R antagonist) or apocynin (a NOX inhibitor) could rescue the abnormal cardiovascular parameters. Both pre- and postnatal-onset treatments with losartan or apocynin corrected the elevated blood pressure levels seen in 16 week-old DD mice (Figure 3A). Blood pressure control was associated with a significant reduction of angII plasma levels in all treated with respect to untreated DD mice, although the levels still remained higher than those of wild-type mice (Figure 3B). Both drugs acted synergistically with the genetic reduction of *Ncf1* gene dosage, as shown by the below normal values of angII in treated DD*/Ncf1*− mice (Table S5). The therapeutic effect was evident at the gene transcription level, since a significant reduction of transcripts encoding three angII biosynthetic pathway proteins was observed both in DD (Figure 3C) and DD*/Ncf1*− mice (Table S6). A reduction in ROS production was also noted in the ascending aortas of treated DD mice (Figure 4A and Table S7), as well as down regulation of several oxidative stress genes, including *Ncf1*, *Ncf2*, *Nox2* and *Nox4* (Figure 4B and Table S8).

**Figure 5:**
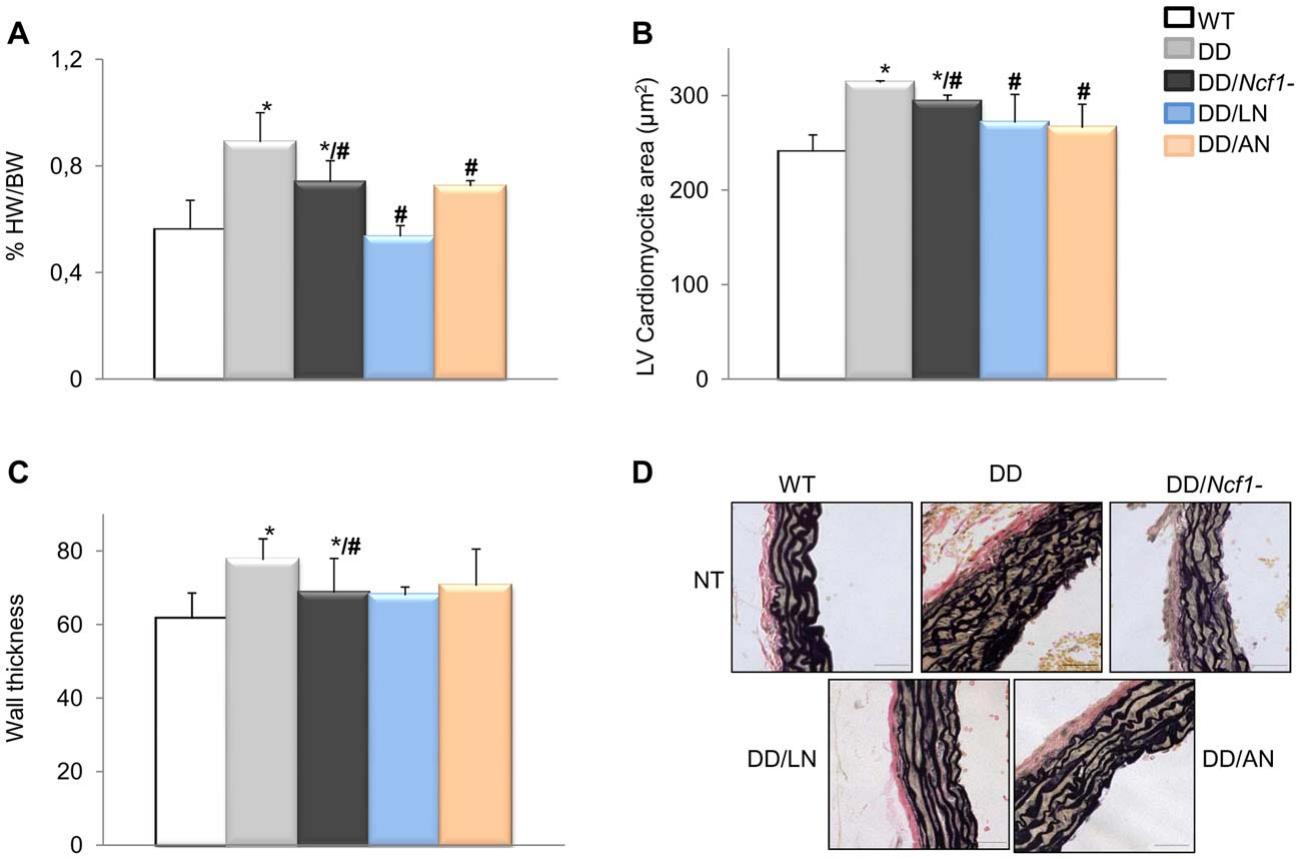
Histopathological analyses of hearts and aortic walls. (A) Treatments with both losartan and apocynin effectively contributed to a normal heart weight relative to body weight, in DD mice. (B) Normal heart weight in treated animals positively correlated with normalization of cardiomyocyte size in both left (LV) and right (RV) ventricles (only represented LV). (C) Average arterial wall size in the ascending aorta of the different groups of animals. Treated DD mice showed slightly but significantly decreased arterial wall thickness when compared to untreated DD animals. (D) Representative axial aortic histological sections from the ascending aorta (40×) with Verhoeff-van Gieson (VVG) staining. The improvement in the organization of elastin sheets is evident in the vessels of treated DD and DD/*Ncf1−* mice when compared to untreated animals. Bars = 200 µm. The results represent the mean ± SD (n = 7–12 per group). Only results from the post-natal onset groups for both treatments are represented. *P<0.05 *versus* wild-type; #*P*<0.05 versus untreated DD mice.

Either apocynin or losartan therapy also completely prevented the cardiac hypertrophy of DD mice. Treated DD animals displayed heart weights and cardiomyocyte sizes similar to those of wild-type counterparts, significantly smaller than those of the untreated DD group (Figure 5A and 5B). A mild improvement of the arterial wall thickness was also evident in all animals treated with both medications, along with reduced elastic fiber fragmentation during histological observation (Figure 5C and 5D). All evaluated parameters of cardiovascular phenotypic rescue (blood pressure, heart size, vascular morphology) persisted at 32 weeks of age in treated mice.

### Secondary effects of pharmacological treatments

We found a high proportion of fetal deaths (∼32%) associated with the prenatal administration of losartan (Table S9). No difference in the expected Mendelian proportions was observed in the offspring of heterozygous crosses (DD x *Ncf1*+/−). We also observed premature postnatal deaths in ∼15% of the treated mice with prenatal-onset losartan (Table S9), mostly due to renal failure before the age of sacrifice, with similar frequencies among genotypes. Our data are in agreement with previous reports contraindicating losartan in pregnancy due to its potential teratogenicity [19], [20]. No specific genotype was associated with increased susceptibility to losartan toxicity. On the other hand, no instances of prenatal death or early postnatal complications were observed in apocynin treated animals, and no other complications were observed in any of the groups treated with postnatal onset.

Note: The full dataset of clinical, morphological, biochemical and molecular parameters at the different time-points, including the effects of treatment on wild-type and DD/*Ncf1*− animals, is provided as supplementary information (Tables S1, S2, S3, S4, S5, S6, S7, S8, S9).

## Discussion

The majority of patients with WBS (84%) manifest cardiovascular problems throughout their lives, particularly an arteriopathy consisting of stenosis of medium and large size arteries that can be present at birth [4]. Hypertension is found in 40%–70% of patients, even during childhood, and there is a significant risk of other cardiovascular complications, such as stroke, cardiac ischemia, and sudden death [4], [6], [8], [21], [22], [23], [24], [25]. Surgical treatment of focal vascular lesions is required in ∼20% of cases and frequently relies on vascular grafts or balloon dilatation angioplasty [4]. Although β-adrenergic blocker and calcium channel blocker drugs have been utilized, there is insufficient evidence to recommend a specific drug therapy for hypertension [8], [22], [26], [27]. Molecules that can either promote elastin biosynthesis or suppress vascular smooth muscle cell proliferation and migration, such as minoxidil [28], glucocorticoids [29], and retinoids [30] have been proposed as possible approaches to the treatment of cardiovascular disease, but none of them have shown to be clinically effective yet. Therefore, additional insight into the pathophysiology is needed to define better-targeted therapies as alternatives to current protocols to prevent the common complications of WBS arteriopathy.

A recently developed mouse model with elastin deficiency (DD) has provided further insight into the cardiovascular disease of WBS [17]. DD mice develop morphological changes in the aortic wall (thickening with disorganized elastin fibers) leading to chronic hypertension and cardiac hypertrophy. As in the case of *Eln* ^+/−^ mice, hypertension of DD mice is related to elevated angII plasma levels, and we have also shown over-expression of several NOX-related genes (*Ncf1*, *Ncf2*, *Nox2/Cybb* and *Nox4*) and significantly increased oxidative stress in these mice. AngII is an important physiological regulator of blood pressure and cardiac function, with hypertensive, growth, and remodeling effects mediated through AT1R. AngII acting through AT1R is also known to stimulate NOX generating ROS in a variety of cells [31]. Chronic infusion of angII in rats increases vascular NOX-derived ROS preceded by a prominent expression of the p47*^phox^* subunit of NOX in the vasculature and kidney [32]. Although some of the ROS serve as signaling molecules in the cells, excessive production is damaging and has been implicated in the progression of many disease processes.

In WBS patients, deletions are almost identical in size and mediated by non allelic homologous recombination, but the deletion breakpoints determine whether a functional copy of the *NCF1* gene is included or not in the deleted interval [33]. Patients with *ELN* deletion and only one functional *NCF1* allele have a 4-fold decreased risk of hypertension compared with those with more than one copy of *NCF1* [15]. Interestingly, mean blood pressure in adult D/P mice, combining DD and PD deletions, was only slightly increased by ∼10%, suggesting a modifying effect of gene(s) within or near the PD region on blood pressure in these mice [11]. In addition, the presence of the PD deletion somehow decreased *Ncf1* expression, being likely the main modifier for the non-significant blood pressure increase in D/P mice. Similarly, by genetic crossing, we have demonstrated that the loss of a functional copy of *Ncf1* in DD mice completely restored oxidative stress and plasma angII levels preventing development of serious cardiovascular anomalies. These data reinforced the idea that pharmacological NOX inhibition could be efficient in the treatment of DD mice. Genetic ablation or pharmacological inhibition of *Nox4* has proven to have a remarkable neuroprotective role in a mouse model for cerebral ischemia [34]. Antioxidants could also decrease blood pressure in several models of hypertension with proven implication of angII and the redox system, acting to scavenge the ROS produced by NOX, but their clinical effectiveness is limited [35].

The AT1R blocker losartan is known to lower blood pressure and rescue vascular wall alterations in other connective tissue defects by inhibiting the TGF-β signaling [14], [36]. Losartan inhibits the growth and remodeling effects of angII but also the NOX generated ROS, all mediated through AT1R.

On the other hand, apocynin is a naturally occurring methoxy-substituted catechol, experimentally used as a more specific inhibitor of NOX with anti-inflammatory activity demonstrated in a variety of cell and animal models. In resting cells, p47^phox^ is folded in on itself through intramolecular interactions between the autoinhibitory region and the bis-SH3 and PX domains. These interactions are destabilized by phosphorylated serine residues within the autoinhibitory region, allowing p47^phox^ to adopt an activated open conformation. Apocynin is thought to inhibit NADPH-oxidase assembly by preventing phosphorylation of the autoinhibitory region of p47^phox^, along with some scavenger activity of hydrogen peroxide [37]. Despite the controversy about the specific mode of action to decrease NOX activity, apocynin has been successfully used in a mouse model to treat hypertension and faster arterial thrombosis [38].

We have evaluated the efficacy and safety of both drugs, losartan and apocynin, in our mouse model of WBS cardiovascular pathology using previously titrated dosages with prenatal and postnatal onset of the therapies [36], [38]. Both treatments were highly effective in the prevention of the development of cardiovascular anomalies in DD mice. Similar effects were manifested by reducing the consequences of NOX activity, with almost complete control of hormonal and biochemical parameters in plasma and tissues, and resulting in normalized blood pressure and improved cardiovascular histology. There was an improvement in aortic wall thickness and architecture without complete reversion of the developmental anomalies secondary to elastin deficiency. Apocynin was as efficient as losartan in prenatal onset, with excellent tolerance and without secondary effects. However, a high proportion of fetal and premature postnatal deaths were associated with the prenatal administration of losartan, supporting its contraindication in pregnancy [19], [20]. Both drugs had significant beneficial effects after postnatal onset of the intervention, with excellent tolerance and no secondary effects.

In conclusion, both, losartan and apocynin, have significant efficacy in the treatment of the cardiovascular phenotype of a mouse model for WBS. Losartan is already approved for human use, while apocynin has been used by inhaler in some clinical trials [39]. The validation of apocynin for human use and the development of additional specific inhibitors of NOX are of great interest, given their potential therapeutic utility in some forms of cardiovascular disease [40]. We believe that these drugs merit evaluation as potential therapeutic agents to prevent the serious cardiovascular problems in human patients with WBS.

## Methods

The study has been performed in accordance with the ARRIVE guidelines, reporting of *in vivo* experiments (http://www.nc3rs.org/ARRIVE).

### Ethics statement

Animal procedures were conducted in strict accordance with the guidelines of the European Communities Directive 86/609/ EEC regulating animal research and were approved by the local Committee of Ethical Animal Experimentation (CEEA-PRBB).

### Animal models

Previously reported mice bearing a heterozygous deletion of half of the orthologous region of the WBS locus on chromosomal band 5G1 (0.67 Mb from *Limk1* to *Trim50*, including *Eln*), called DD for distal deletion, were used as a model for the WBS cardiovascular phenotype [11], [17]. Mice with the proximal half-deletion of the orthologous WBS locus (0.45 Mb from *Limk1* to *Gtf1i*), called PD, and the double mutants *in trans* (with homozygous *Limk1* deletion), D/P [17] were also used for some studies. Heterozygous DD animals were crossed with mice bearing a homozygous loss of function mutation of the *Ncf1* gene (B6 (Cg)-Ncf1m1J)[18] to obtain double mutants in the first generation (DD*/Ncf1*−), harbouring then a mutant allele (DD deletion and *Ncf1* mutation) in each chromosome (Figure 1A). All mice were bred on a majority C57BL/6J background (97%). Tail clipping was performed within 4 weeks of birth to determine the genotype of each mouse using PCR and appropriate primers (See primer sequences in Table S10).

### Animal treatments

Fifteen different groups of mice (7–15 littermate animals per group, 5 groups per genotype: wild-type, DD or DD*/Ncf1*−), were used in this study for a total of n = 208. The 5 groups per genotype corresponded to untreated animals (NT), treated with losartan (Coozar, MSD) with prenatal (LP) or postnatal onset (LN), and treated with apocynin (Sigma) with prenatal (AP) or postnatal onset (AN). As previously described, drugs were administered in the drinking water with final concentrations of 0.002 g/day for losartan [36] and 2.5×10^−4^ g/day for apocynin [41]. In the groups of prenatal initiation, pregnant females started treatment at 14.5 dpc and therapy was continued throughout lactation. Postnatal treatments started at 7 weeks of age. In both cases, mice continued on oral therapy until 16 or 32 weeks of age, when they were sacrificed. Drinking water with drugs were refreshed every 3 days and protected from light by wrapping the drinking water container with aluminum foil. We recorded drinking volumes for untreated and treated mice in order to avoid any interference in the drinking water because of drugs supplement (Table S11).

### Blood pressure measurements

Systolic, mean, and diastolic blood pressure were measured in conscious male mice on three separate occasions by using a tail cuff system (Non-Invasive Blood Pressure System, PanLab), while holding the mice in a black box on a heated stage. In order to improve measurement consistency, multiple sessions were performed to train each mouse. At least 12 readings (4 per session) were made for each mouse (n = 7–15 per group).

### Histopathology

Animals were sacrificed at two time points (16 or 32-week-old). Immediately following sacrifice, all the organs in the thoracic cage (thymus, lung, heart and aorta) were removed in block and fixed in 10% buffered formalin at 4°C for 16 hours. Hearts and aorta were dissected, washed, and weighed (wet weight). Hearts and vessels were processed for paraffin embedding. Wall thickness and lamellar units were analyzed using 5 µm cross-sections of the ascending aorta (transected immediately below the level of the brachiocephalic artery) stained with Verhoeff-van Gieson (VVG) to visualize elastic lamina. Wall thickness at 10 different representative locations was measured and averaged by an observer blinded to genotype and treatment arm for each mouse. The number of medial lamellar units (MLUs) at 4 sites was assessed and averaged by 2 separate blinded observers. These axial cross-sections were imaged with an Olympus BXS1 microscope with epifluorescence and phase-contrast optics equipped with the Olympus DP71 camera, and images were captured with the Cell^B^ Digital Imaging system software. MLUs counting and wall thickness were quantified using Adobe Photoshop CS (Adobe Systems).

### Quantification of mRNA

RNA was extracted from the visceral organs of the thorax by using TRIZOL reagent (Invitrogen) according to the manufacturer's instructions, followed by a second spin columns (Qiagen) purification. To avoid possible contamination of gDNA, all samples were analyzed before conversion to cDNA using standard PCR. In addition, primers were designed in different exons to avoid undesired amplification. cDNA was prepared from 1 µg total RNA using random hexamers and SuperScript II RNase H- reverse transcriptase (Invitrogen). The expression of genes involved in the angII biosynthetic pathway (*Agt*, *Ren* and *Ace*) and NOX-related oxidative stress (*Ncf1*, *Ncf2*, *Nox2/Cybb*, *Nox4*, *Cyba* and *Rac2*) were evaluated by quantitative real-time PCR (qRT-PCR). After diluting the cDNA (from 1∶10 to 1∶100, depending on the tissue), 5 µl were used as template for qRT-PCR using an ABI5700 thermocycler (Applied Biosystems) with the FastStart DNA Master SYBR Green Kit (Roche) and gene specific primers. Characteristics of primers are given in Table S10. Amplification of the *Rps28* transcript served as RNA control for relative quantification. Each sample and the corresponding negative controls for each pair of primers were analyzed in triplicate at least in two independent experiments. Threshold cycle values were set manually and analyzed using the comparative method [42].

### Measurements of plasma angII levels

Blood was collected from the mouse heart into EDTA tubes immediately after sacrifice. Plasma was collected after centrifugation at 1,500 g for 10 minutes and stored at −80°C until use. AngII levels were determined with the Renin Fluorometric Assay Kit Sensolyte 520 following the manufacturer's instructions.

### Superoxide anion and nitrotyrosine detection

Formalin-fixed, paraffin-embedded transverse sections (5 µm in thickness) were mounted on polylysine-coated glass slides. After blockade with 5% bovine serum albumin plus 0.1% Triton X-100 in phosphate-buffered saline overnight at 4°C, the sections were incubated for 90 min at 37°C with the fluorescent probe DHE (Calbiochem, Darmstadt, Germany). In the presence of O2-, DHE is oxidized to ethidium, which intercalates with DNA, and yields bright red fluorescence. After washing with PBS plus 0.1% Triton X-100, sections were mounted and visualized by fluorescence microscopy (Olympus BX51, Japan). DHE fluorescence intensity was analyzed with NIH ImageJ software (v1.43, April 2010;U.S. National Institutes of Health, Bethesda, MD) as previously described [43]. The fluorescence intensity is proportional to the amount of superoxide anion. Thereafter, sections were incubated with 40,6-diamindino-2-fenilindol (DAPI) (300 nM) for 5 min at 37°C, reactive with fluorescent blue, marking the interlayer between DNA base pairs of cell nuclei. DAPI staining of cell nuclei helps detect true DHE staining (present in the nucleus) versus nonspecific staining. The specificity of the immunostaining was evaluated by the omission of the dye (negative controls). For the quantification of fluorescence, we also subtracted the background present in the negative control, in an attempt to eliminate any autofluorescence. All comparisons were made on cuts prepared with the same experimental conditions and the same day.

The distribution of 3-nitrotyrosine residues, as an indirect marker of peroxynitrite (ONOO-) production, was evaluated by indirect immunofluorescence. In brief, arterial sections were blocked for 2 h at 37°C and incubated overnight at 4°C with a polyclonal anti-nitrotyrosine antibody (dilution 1∶100; Chemicon International, Temecula, CA, USA).

### Statistics

All data are presented as means ± SD. Statistical analysis was performed using ANOVA with a post hoc Bonferroni comparison between multiple groups. In specific cases of two-group comparisons we performed t-test. Values of p<0.05 were considered significant.

## Supporting Information

Figure S1 Relative transcript levels of the *Ace*, *Agt*, and *Ren* genes in several tissues of the DD and DD/*Ncf1* mice. A significantly increased expression of the three genes was observed in all tissues tested of DD mice. DD/*Ncf1* mice showed slightly increased *Ace* and *Agt* expression, significantly lower than DD animals, and normal *Ren* expression in all tissues. Data were normalized such that the mean of the wild-type group was 1.0 represented by the blue line. *P<0.05 versus wild-type.; #*P*<0.05 versus DD mice. The results represent the mean ± SD (n = 5–7 per group).
(TIF)

Figure S2 Relative transcript levels of the *Ncf2*, *Nox2*, *Nox4* and *Cyba* genes in heart, aorta, and lung of PD, DD, and D/P mice. Significantly increased expression of three genes (*Ncf2*, *Nox2* and *Nox4*) is noted in all tissues of DD mice, with *Cyba* expression also increased in lung. Elevated expression of *Nox2* was also observed in all tissues of D/P animals, along with mild elevation of *Nox4* and *Cyba* in the aorta. Data were normalized such that the mean of the wild-type group was 1.0 represented by the blue line. *P<0.05 versus wild-type.; #*P*<0.05 versus untreated DD mice. The results represent the mean ± SD (n = 3–4 per group).
(TIF)

Table S1 Blood pressure measurements at 16 weeks of age. Systolic, diastolic and mean blood pressure (Figure 2A and Figure 3A) were recorded “in vivo” from 16-weeks-old mice. Mean and SD values of the different groups according to each genotype and intervention are shown. Statistical analysis was done using ANOVA with a post hoc Bonferroni comparison among multiple groups. *P*-values of the different comparisons are also shown, with significant values displayed in bold. WT: wild-type; DD: distal deletion; DD/*Ncf1*−: double heterozygous for DD and *Ncf1* (*in trans*); NT: no treatment; LN: losartan postnatal; LP: losartan prenatal; AN: apocynin postnatal; AP: apocynin prenatal.
(PDF)

Table S2 Mean blood pressure at 8 and 32 weeks. Mean blood pressure was recorded “in vivo” from 8 and 32-weeks-old mice. Mean and SD values of the different groups according to each genotype and intervention are shown. Statistical analysis was done using ANOVA with a post hoc Bonferroni comparison among multiple groups. *P*-values of the different comparisons are also shown, with significant values displayed in bold. WT: wild-type; DD: distal deletion; DD/*Ncf1*−: double heterozygous for DD and *Ncf1* (*in trans*); NT: no treatment; LN: losartan postnatal; LP: losartan prenatal; AN: apocynin postnatal; AP: apocynin prenatal.
(PDF)

Table S3 Histopathology at 16 weeks. Histological parameters of the cardiovascular system recorded in 16-weeks-old mice after sacrifice; including the aortic wall thickness, the number of lamellar units in the aortic wall, the proportion of heart weight versus body weight and the cross sectional area of cardiomyocytes in the left and right ventricles (Figure 2D, 2E and Figure 5). Mean and SD values of the different groups according to each genotype and intervention are shown. Statistical analysis was done using ANOVA with a post hoc Bonferroni comparison among multiple groups. *P*-values of the different comparisons are also shown, with significant values displayed in bold. WT: wild-type; DD: distal deletion; DD/*Ncf1*−: double heterozygous for DD and *Ncf1* (*in trans*); NT: no treatment; LN: losartan postnatal; LP: losartan prenatal; AN: apocynin postnatal; AP: apocynin prenatal.
(PDF)

Table S4 Histopathology at 32 weeks. Histological parameters of the cardiovascular system recorded in 32-weeks-old mice after sacrifice, including the aortic wall thickness, the number of lamellar units in the aortic wall and the proportion of heart weight versus body weight. Mean and SD values of the different groups according to each genotype and intervention are shown. Statistical analysis was done using ANOVA with a post hoc Bonferroni comparison among multiple groups. *P*-values of the different comparisons are also shown, with significant values displayed in bold. WT: wild-type; DD: distal deletion; DD/*Ncf1*−: double heterozygous for DD and *Ncf1* (*in trans*); NT: no treatment; LN: losartan postnatal; LP: losartan prenatal; AN: apocynin postnatal; AP: apocynin prenatal.
(PDF)

Table S5 AngII levels in plasma. AngII plasma levels were recorded in mice 16 and 32-weeks-old (Figure 2B and Figure 3B). Mean and SD values of the different groups according to each genotype and intervention are shown. Statistical analyses were done using ANOVA with a post hoc Bonferroni comparison among multiple groups. *P*-values of the different comparisons are also shown, with significant values displayed in bold. WT: wild-type; DD: distal deletion; DD/*Ncf1*−: double heterozygous for DD and *Ncf1* (*in trans*); NT: no treatment; LT: losartan treatment; AT: apocynin treatment. Data in the groups with prenatal and postnatal onset for both drugs were similar and have been joined as single groups.
(PDF)

Table S6 Relative mRNA levels of angII/Ren pathway genes in different tissues. Mean and SD values of mRNA levels of angII/renin pathway genes recorded by qRT-PCR analysis in heart, aorta and lung of 16-weeks-old mice (Figure 2C, Figure 3C and Figure S1) and mean and SD values of mRNA levels of Ren gene recorded by qRT-PCR analysis in kidneys of 16-weeks-old mice (Figure S1). Each sample and the corresponding negative controls for each pair of primers were analyzed in triplicate at least in two independent experiments. Statistical analyses of two-group comparisons were performed by t-test. *P*-values of the different comparisons are also shown, with significant values displayed in bold. WT: wild-type; DD: distal deletion; DD/*Ncf1*−: double heterozygous for DD and *Ncf1* (*in trans*). NT: no treatment; LP: losartan prenatal; AP: apocynin prenatal onset treatments.
(PDF)

Table S7 Quantification of protein nitrosylation and DHE fluorescence in the aortic wall. Mean and SD values of fluorescence intensity per area in the different groups of genotypes and interventions are shown, expressed as arbitrary units of fluorescence (Figure 2F and Figure 4A, 4B). Background effect was minimized by subtraction of the values obtained in negative control samples (no DHE). All aortic wall sections studied were prepared simultaneously and using identical experimental conditions to avoid experimental biases. Statistical analyses were done using ANOVA with a post hoc Bonferroni comparison among multiple groups. *P*-values of the different comparisons are also shown, with significant values displayed in bold. WT: wild-type; DD: distal deletion; DD/*Ncf1*−: double heterozygous for DD and *Ncf1* (*in trans*); NT: no treatment; LN: losartan postnatal; LP: losartan prenatal; AN: apocynin postnatal; AP: apocynin prenatal.
(PDF)

Table S8 Relative mRNA levels of oxidative stress molecules in heart, aorta and lung. Relative mRNA levels (mean and SD values) of 5 NOX-related genes were recorded by qRT-PCR analysis in hearts, aortas and lungs (Figure 1B, 1C and Figure 4B) of 16-weeks-old mice. Each sample and the corresponding negative control for each pair of primers were analyzed in triplicate at least in two independent experiments. Statistical analyses with two-group comparisons were performed by t-test. *P*-values of the different comparisons are also shown, with significant values displayed in bold. DD: distal deletion (from *Limk1* to *Trim50*); PD: proximal deletion (from *Gtf2i* to *Limk1*); D/P: double heterozygous for DD and PD deletions (*in trans*). NT: no treatment; LT: losartan treatment; AT: apocynin treatment.
(PDF)

Table S9 Secondary effects of pharmacological treatments. Prenatal outcomes and premature postnatal deaths in treated animals. We found a high proportion of fetal deaths (∼32%) only associated with the prenatal administration of losartan, calculated by the expected number of pups born by mate. No differences among genotypes were observed.
(PDF)

Table S10 Primer sequences and PCR conditions for qRT-PCR and genotyping. The locus name, primer sequences, amplicon size, genomic location and the optimal melting temperatures for each specific primer are shown. Conditions for qRT-PCR are also shown, following the recommendations of the MIQE guidelines.
(PDF)

Table S11 Recording of drinking volumes. We recorded daily drinking volumes of all untreated and treated mice. The table displays the mean daily volume drank per animal in ml. Up to 4 littermate animals were stocked per cage, regardless of their genotype. No significant differences between groups were observed. NT: no treatment; LN: losartan postnatal treatment; AN: apocynin postnatal treatment.
(PDF)
