# Supplementary figures and images for: Reduction of NADPH-Oxidase Activity Ameliorates the Cardiovascular Phenotype in a Mouse Model of Williams-Beuren Syndrome
Source: PLoS Genet. 2012 Feb 2;8(2):e1002458. doi: 10.1371/journal.pgen.1002458 (PMC3271062; doi:10.1371/journal.pgen.1002458)

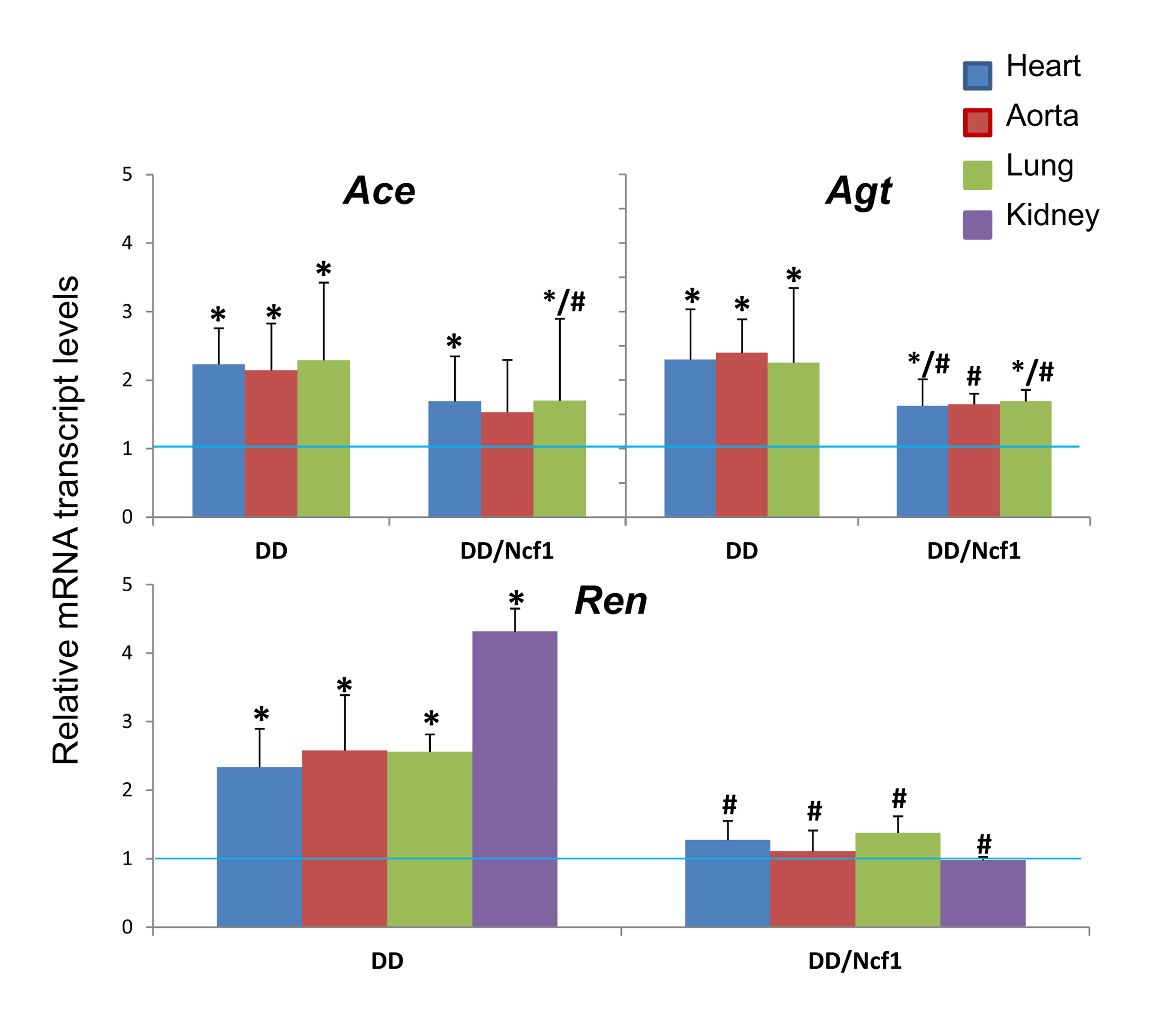

Supplement: Figure S1 — Relative transcript levels of the Ace, Agt, and Ren genes in several tissues of the DD and DD/Ncf1 mice. A significantly increased expression of the three genes was observed in all tissues tested of DD mice. DD/Ncf1 mice showed slightly increased Ace and Agt expression, significantly lower than DD animals, and normal Ren expression in all tissues. Data were normalized such that the mean of the wild-type group was 1.0 represented by the blue line. *P<0.05 versus wild-type.; #P<0.05 versus DD mice. The results represent the mean ± SD (n = 5–7 per group). (TIF) [file pgen.1002458.s001.tif]

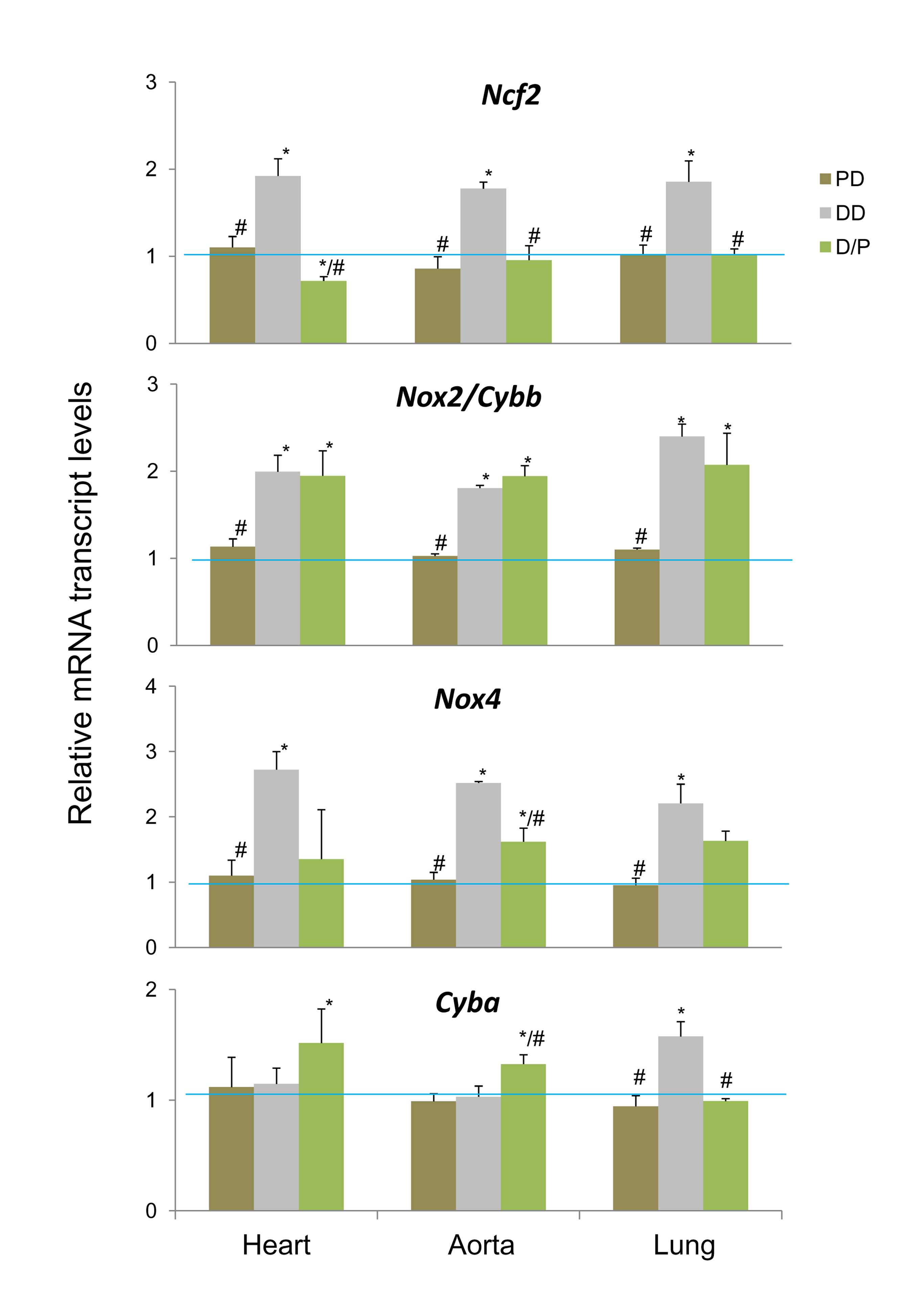

Supplement: Figure S2 — Relative transcript levels of the Ncf2, Nox2, Nox4 and Cyba genes in heart, aorta, and lung of PD, DD, and D/P mice. Significantly increased expression of three genes (Ncf2, Nox2 and Nox4) is noted in all tissues of DD mice, with Cyba expression also increased in lung. Elevated expression of Nox2 was also observed in all tissues of D/P animals, along with mild elevation of Nox4 and Cyba in the aorta. Data were normalized such that the mean of the wild-type group was 1.0 represented by the blue line. *P<0.05 versus wild-type.; #P<0.05 versus untreated DD mice. The results represent the mean ± SD (n = 3–4 per group). (TIF) [file pgen.1002458.s002.tif]
