# Supplementary material for: Reduction of NADPH-Oxidase Activity Ameliorates the Cardiovascular Phenotype in a Mouse Model of Williams-Beuren Syndrome
Source: PLoS Genet. 2012 Feb 2;8(2):e1002458. doi: 10.1371/journal.pgen.1002458 (PMC3271062; doi:10.1371/journal.pgen.1002458)
Supplement: Table S1 — Blood pressure measurements at 16 weeks of age. Systolic, diastolic and mean blood pressure (Figure 2A and Figure 3A) were recorded “in vivo” from 16-weeks-old mice. Mean and SD values of the different groups according to each genotype and intervention are shown. Statistical analysis was done using ANOVA with a post hoc Bonferroni comparison among multiple groups. P-values of the different comparisons are also shown, with significant values displayed in bold. WT: wild-type; DD: distal deletion; DD/Ncf1−: double heterozygous for DD and Ncf1 (in trans); NT: no treatment; LN: losartan postnatal; LP: losartan prenatal; AN: apocynin postnatal; AP: apocynin prenatal. (PDF) [file pgen.1002458.s003.pdf]

Table S1: Blood pressure measurements at 16 weeks of age (N=7-12)

SYSTOLIC BLOOD PRESSURE

Genotype	Intervention	SYS-mean	SD	*P vs WT-NT*	*P vs DD-NT*
WT	NT	114.58	7.68		
WT	LP	104.90	3.59	1.000	
WT	AP	103.11	8.76	1.000	
WT	LN	105.40	11.97	1.000	
WT	AN	103.72	8.62	1.000	
DD	NT	151.43	12.64	**0.000**	
DD	LP	109.88	12.77	1.000	**0.000**
DD	AP	120.23	12.89	1.000	**0.001**
DD	LN	113.23	16.03	1.000	**0.002**
DD	AN	120.23	7.93	0.752	**0.008**
DD/*Ncf1-*	NT	121.83	9.33	0.805	**0.000**
DD/*Ncf1-*	LP	112.07	10.35	1.000	**0.000**
DD/*Ncf1-*	AP	115.31	10.65	1.000	**0.000**
DD/*Ncf1-*	LN	113.26	20.32	1.000	**0.001**
DD/*Ncf1-*	AN	116.84	5.99	1.000	**0.000**

DIASTOLIC BLOOD PRESSURE

Genotype	Intervention	DIAS-mean	SD	*P vs WT-NT*	*P vs DD-NT*
WT	NT	87.80	8.89		
WT	LP	85.60	5.23	1.000	
WT	LN	85.30	8.03	1.000	
WT	AP	81.85	6.70	1.000	
WT	AN	81.63	6.47	1.000	
DD	NT	129.53	10.08	**0.000**	
DD	LP	93.18	10.84	1.000	**0.001**
DD	LN	90.57	14.16	1.000	**0.000**
DD	AP	95.83	12.35	0.636	**0.000**
DD	AN	97.20	10.71	0.481	**0.002**
DD/*Ncf1-*	NT	93.54	8.67	0.974	**0.000**
DD/*Ncf1-*	LP	86.69	9.18	1.000	**0.000**
DD/*Ncf1-*	LN	90.27	11.72	1.000	**0.000**
DD/*Ncf1-*	AP	90.00	8.77	1.000	**0.000**
DD/*Ncf1-*	AN	95.64	8.85	0.575	**0.000**

MEAN BLOOD PRESSURE

Genotype	Intervention	MEAN-mean	s.e.m.	*P vs WT-NT*	*P vs DD-NT*
WT	NT	96.18	8.08		
WT	LP	90.75	3.69	1.000	
WT	LN	92.15	8.52	1.000	
WT	AP	89.22	7.39	1.000	
WT	AN	88.70	6.85	1.000	
DD	NT	135.94	10.78	**0.000**	
DD	LP	97.56	11.57	1.000	**0.000**
DD	LN	98.10	13.47	1.000	**0.000**
DD	AP	103.50	11.70	0.705	**0.000**

Table S1: Blood pressure measurements at 16 weeks of age (N=7-12)

DD	AN	104.61	9.62	0.466	**0.003**
DD/*Ncf1*-	NT	101.09	7.57	0.654	**0.000**
DD/*Ncf1*-	LP	94.67	8.41	1.000	**0.000**
DD/*Ncf1*-	LN	98.01	14.22	1.000	**0.000**
DD/*Ncf1*-	AP	98.16	9.18	1.000	**0.000**
DD/*Ncf1*-	AN	102.54	7.55	0.466	**0.000**
