# Supplementary material for: Reduction of NADPH-Oxidase Activity Ameliorates the Cardiovascular Phenotype in a Mouse Model of Williams-Beuren Syndrome
Source: PLoS Genet. 2012 Feb 2;8(2):e1002458. doi: 10.1371/journal.pgen.1002458 (PMC3271062; doi:10.1371/journal.pgen.1002458)
Supplement: Table S2 — Mean blood pressure at 8 and 32 weeks. Mean blood pressure was recorded “in vivo” from 8 and 32-weeks-old mice. Mean and SD values of the different groups according to each genotype and intervention are shown. Statistical analysis was done using ANOVA with a post hoc Bonferroni comparison among multiple groups. P-values of the different comparisons are also shown, with significant values displayed in bold. WT: wild-type; DD: distal deletion; DD/Ncf1−: double heterozygous for DD and Ncf1 (in trans); NT: no treatment; LN: losartan postnatal; LP: losartan prenatal; AN: apocynin postnatal; AP: apocynin prenatal. (PDF) [file pgen.1002458.s004.pdf]

Table S2: Mean blood pressure at 8 and 32 weeks of age

8 weeks (N=7-12)

Genotype	Intervention	Mean	SD	*P vs WT-NT*	*P vs DD-NT*
WT	NT	91.08	8.99		
WT	LP	78.25	5.91	0.088	
WT	AP	89.94	8.45	1.000	
DD	NT	126.13	9.51	**0.000**	
DD	LP	100.90	9.10	0.225	**0.000**
DD	AP	106.78	6.01	**0.036**	**0.001**
DD/*Ncf1-*	NT	106.07	9.77	**0.001**	**0.000**
DD/*Ncf1-*	LP	98.00	9.54	0.371	**0.000**
DD/*Ncf1-*	AP	98.87	6.81	0.260	**0.000**

32 weeks (N=3-7)

Genotype	Intervention	Mean	SD	*P vs WT-NT*	*P vs DD-NT*
WT	NT	93.71	6.58		
WT	LP	81.06	1.88	**0.013**	
WT	LN	91.36	6.81	1.000	
WT	AP	95.84	7.95	1.000	
WT	AN	87.30	1.65	0.595	
DD	NT	138.15	15.94	**0.000**	
DD	LP	93.21	15.44	1.000	**0.001**
DD	LN	102.30	3.90	0.064	**0.004**
DD	AP	101.68	13.62	0.709	**0.010**
DD	AN	101.95	6.72	0.227	**0.005**
DD/*Ncf1-*	NT	104.67	9.88	0.109	**0.000**
DD/*Ncf1-*	LP	96.60	6.41	1.000	**0.000**
DD/*Ncf1-*	LN	91.27	7.05	1.000	**0.000**
DD/*Ncf1-*	AP	96.03	15.45	1.000	**0.000**
DD/*Ncf1-*	AN	86.69	9.56	0.727	**0.000**
