# Supplementary material for: Reduction of NADPH-Oxidase Activity Ameliorates the Cardiovascular Phenotype in a Mouse Model of Williams-Beuren Syndrome
Source: PLoS Genet. 2012 Feb 2;8(2):e1002458. doi: 10.1371/journal.pgen.1002458 (PMC3271062; doi:10.1371/journal.pgen.1002458)
Supplement: Table S3 — Histopathology at 16 weeks. Histological parameters of the cardiovascular system recorded in 16-weeks-old mice after sacrifice; including the aortic wall thickness, the number of lamellar units in the aortic wall, the proportion of heart weight versus body weight and the cross sectional area of cardiomyocytes in the left and right ventricles (Figure 2D, 2E and Figure 5). Mean and SD values of the different groups according to each genotype and intervention are shown. Statistical analysis was done using ANOVA with a post hoc Bonferroni comparison among multiple groups. P-values of the different comparisons are also shown, with significant values displayed in bold. WT: wild-type; DD: distal deletion; DD/Ncf1−: double heterozygous for DD and Ncf1 (in trans); NT: no treatment; LN: losartan postnatal; LP: losartan prenatal; AN: apocynin postnatal; AP: apocynin prenatal. (PDF) [file pgen.1002458.s005.pdf]

Table S3: Histopathology at 16 weeks of age

Aortic wall thickness

Genotype	Intervention	Mean	SD	*P vs WT-NT*	*P vs DD-NT*
WT	NT	61.61	6.98		
WT	LP	57.84	10.17	1.000	
WT	LN	58.88	8.44	1.000	
WT	AP	64.91	10.34	1.000	
WT	AN	61.50	4.44	1.000	
DD	NT	77.61	5.70	**0.000**	
DD	LP	58.39	18.43	1.000	**0.008**
DD	LN	68.02	2.19	0.376	1.000
DD	AP	70.74	8.06	0.069	1.000
DD	AN	72.11	8.64	0.078	1.000
DD/*Ncf1*-	NT	68.69	9.29	**0.035**	**0.007**
DD/*Ncf1*-	LP	62.64	10.35	1.000	**0.002**
DD/*Ncf1*-	LN	68.72	5.23	0.125	**0.031**
DD/*Ncf1*-	AP	63.46	3.25	1.000	**0.000**
DD/*Ncf1*-	AN	65.19	10.65	1.000	**0.022**

Number of lamellar units

Genotype	Mean	SD	*P vs WT-NT*
WT	7.12	0.70	
DD	8.39	1.38	**0.016**
DD/*Ncf1*-	7.85	1.31	0.252

% Heart weight / Body weight

Genotype	Intervention	Mean	SD	*P vs WT-NT*	*P vs DD-NT*
WT	NT	0.56	0.11		
WT	LP	0.52	0.07	1.000	
WT	LN	0.46	0.07	0.913	
WT	AP	0.60	0.09	1.000	
WT	AN	0.54	0.11	1.000	
DD	NT	0.89	0.11	**0.000**	
DD	LP	0.65	0.09	0.716	**0.001**
DD	LN	0.54	0.04	1.000	**0.000**
DD	AP	0.69	0.09	0.145	**0.002**
DD	AN	0.73	0.02	0.291	**0.031**
DD/*Ncf1*-	NT	0.74	0.08	**0.046**	**0.039**
DD/*Ncf1*-	LP	0.48	0.10	0.737	**0.000**
DD/*Ncf1*-	LN	0.56	0.07	1.000	**0.000**
DD/*Ncf1*-	AP	0.54	0.07	1.000	**0.000**
DD/*Ncf1*-	AN	0.61	0.16	1.000	**0.003**

Table S3: Histopathology at 16 weeks of age

Cross-sectional area of cardiomyocytes (µm^2)

Left Ventricle (LV)

Genotype	Intervention	Mean	SD	*P vs WT-NT*	*P vs DD-NT*
WT	NT	240.70	17.70		
WT	LP	257.65	33.47	1.000	
WT	LN	253.04	23.61	1.000	
WT	AP	261.95	32.80	1.000	
WT	AN	260.32	33.78	1.000	
DD	NT	313.52	2.12	**0.000**	
DD	LP	260.62	26.52	0.517	**0.006**
DD	LN	271.63	29.57	0.080	**0.046**
DD	AP	267.86	23.09	0.096	**0.023**
DD	AN	266.17	24.60	0.088	**0.017**
DD/*Ncf1-*	NT	294.38	6.17	**0.000**	**0.025**
DD/*Ncf1-*	LP	246.33	26.87	1.000	**0.000**
DD/*Ncf1-*	LN	261.69	15.42	0.348	**0.000**
DD/*Ncf1-*	AP	269.80	18.48	0.069	**0.000**
DD/*Ncf1-*	AN	261.59	9.43	0.201	**0.000**

Right Ventricle (RV)

Genotype	Intervention	Mean	SD	*P vs WT-NT*	*P vs DD-NT*
WT	NT	322.08	18.04		
WT	LP	315.12	25.12	1.000	
WT	LN	327.55	27.74	1.000	
WT	AP	326.17	21.67	1.000	
WT	AN	309.57	17.58	1.000	
DD	NT	433.29	20.24	**0.000**	
DD	LP	344.02	11.22	0.247	**0.000**
DD	LN	340.09	35.21	0.895	**0.000**
DD	AP	364.89	38.51	**0.031**	**0.002**
DD	AN	351.54	18.35	0.100	**0.000**
DD/*Ncf1-*	NT	407.02	6.74	**0.000**	**0.039**
DD/*Ncf1-*	LP	331.18	28.27	1.000	**0.000**
DD/*Ncf1-*	LN	327.52	30.79	1.000	**0.000**
DD/*Ncf1-*	AP	337.36	10.67	0.938	**0.000**
DD/*Ncf1-*	AN	330.09	27.54	1.000	**0.000**
