# Supplementary material for: Reduction of NADPH-Oxidase Activity Ameliorates the Cardiovascular Phenotype in a Mouse Model of Williams-Beuren Syndrome
Source: PLoS Genet. 2012 Feb 2;8(2):e1002458. doi: 10.1371/journal.pgen.1002458 (PMC3271062; doi:10.1371/journal.pgen.1002458)
Supplement: Table S4 — Histopathology at 32 weeks. Histological parameters of the cardiovascular system recorded in 32-weeks-old mice after sacrifice, including the aortic wall thickness, the number of lamellar units in the aortic wall and the proportion of heart weight versus body weight. Mean and SD values of the different groups according to each genotype and intervention are shown. Statistical analysis was done using ANOVA with a post hoc Bonferroni comparison among multiple groups. P-values of the different comparisons are also shown, with significant values displayed in bold. WT: wild-type; DD: distal deletion; DD/Ncf1−: double heterozygous for DD and Ncf1 (in trans); NT: no treatment; LN: losartan postnatal; LP: losartan prenatal; AN: apocynin postnatal; AP: apocynin prenatal. (PDF) [file pgen.1002458.s006.pdf]

Table S4: Histopathology at 32 weeks of age

Aortic wall thickness

Genotype	Intervention	Mean	SD	*P vs WT-NT*	*P vs DD-NT*
WT	NT	66.60	6.78		
WT	LP	57.60	4.75	0.361	
WT	LN	65.08	11.30	1.000	
WT	AP	66.86	9.85	1.000	
WT	AN	67.33	9.94	1.000	
DD	NT	90.45	4.53	**0.000**	
DD	LP	71.43	5.40	0.751	**0.004**
DD	LN	75.42	8.66	0.249	**0.011**
DD	AP	75.03	7.19	0.215	**0.025**
DD	AN	76.03	12.25	0.372	0.084
DD/*Ncf1*-	NT	75.18	3.52	**0.001**	**0.001**
DD/*Ncf1*-	LP	62.52	7.47	0.884	**0.000**
DD/*Ncf1*-	LN	64.38	11.35	1.000	**0.000**
DD/*Ncf1*-	AP	69.56	8.13	1.000	**0.000**
DD/*Ncf1*-	AN	69.99	9.91	1.000	**0.000**

Number of lamellar units

Genotype	Mean	SD	*P vs WT*
WT	7.44	1.81	
DD	8.38	1.77	0.73
DD/*Ncf1* -	7.50	1.07	1.00

Table S4: Histopathology at 32 weeks of age

% Heart weight / Body weight

**Genotype**	**Intervention**	**Mean**	**SD**	***P vs WT-NT***	***P vs DD-NT***
WT	NT	0.59	0.08		
WT	LP	0.51	0.11	1.000	
WT	LN	0.53	0.07	1.000	
WT	AP	0.52	0.13	1.000	
WT	AN	0.58	0.15	0.247	
DD	NT	0.88	0.12	**0.000**	
DD	LP	0.63	0.12	1.000	**0.006**
DD	LN	0.63	0.06	1.000	**0.001**
DD	AP	0.72	0.10	**0.036**	**0.048**
DD	AN	0.75	0.05	0.090	0.123
DD/*Ncf1-*	NT	0.68	0.10	0.140	**0.001**
DD/*Ncf1-*	LP	0.60	0.09	1.000	**0.000**
DD/*Ncf1-*	LN	0.63	0.18	1.000	**0.000**
DD/*Ncf1-*	AP	0.57	0.13	1.000	**0.000**
DD/*Ncf1-*	AN	0.56	0.17	1.000	**0.000**
