# Supplementary material for: Reduction of NADPH-Oxidase Activity Ameliorates the Cardiovascular Phenotype in a Mouse Model of Williams-Beuren Syndrome
Source: PLoS Genet. 2012 Feb 2;8(2):e1002458. doi: 10.1371/journal.pgen.1002458 (PMC3271062; doi:10.1371/journal.pgen.1002458)
Supplement: Table S5 — AngII levels in plasma. AngII plasma levels were recorded in mice 16 and 32-weeks-old (Figure 2B and Figure 3B). Mean and SD values of the different groups according to each genotype and intervention are shown. Statistical analyses were done using ANOVA with a post hoc Bonferroni comparison among multiple groups. P-values of the different comparisons are also shown, with significant values displayed in bold. WT: wild-type; DD: distal deletion; DD/Ncf1−: double heterozygous for DD and Ncf1 (in trans); NT: no treatment; LT: losartan treatment; AT: apocynin treatment. Data in the groups with prenatal and postnatal onset for both drugs were similar and have been joined as single groups. (PDF) [file pgen.1002458.s007.pdf]

Table S5: AngII levels in plasma

**Genotype**	**Intervention**	**Mean**	**SD**	***P vs WT-NT***	***P vs DD-NT***
**WT**	**NT**	48.27	6.67		
**WT**	**LT**	25.30	10.32	0.000	
**WT**	**AT**	25.88	8.17	0.000	
**DD**	**NT**	82.24	7.39	**0.000**	
**DD**	**LT**	61.84	7.44	**0.017**	**0.002**
**DD**	**AT**	66.54	9.31	**0.000**	**0.023**
**DD/*Ncf1-***	**NT**	66.95	7.18	**0.002**	**0.036**
**DD/*Ncf1-***	**LT**	35.81	8.85	**0.011**	**0.000**
**DD/*Ncf1-***	**AT**	46.36	5.21	1.000	**0.000**
