# Supplementary material for: Reduction of NADPH-Oxidase Activity Ameliorates the Cardiovascular Phenotype in a Mouse Model of Williams-Beuren Syndrome
Source: PLoS Genet. 2012 Feb 2;8(2):e1002458. doi: 10.1371/journal.pgen.1002458 (PMC3271062; doi:10.1371/journal.pgen.1002458)
Supplement: Table S6 — Relative mRNA levels of angII/Ren pathway genes in different tissues. Mean and SD values of mRNA levels of angII/renin pathway genes recorded by qRT-PCR analysis in heart, aorta and lung of 16-weeks-old mice (Figure 2C, Figure 3C and Figure S1) and mean and SD values of mRNA levels of Ren gene recorded by qRT-PCR analysis in kidneys of 16-weeks-old mice (Figure S1). Each sample and the corresponding negative controls for each pair of primers were analyzed in triplicate at least in two independent experiments. Statistical analyses of two-group comparisons were performed by t-test. P-values of the different comparisons are also shown, with significant values displayed in bold. WT: wild-type; DD: distal deletion; DD/Ncf1−: double heterozygous for DD and Ncf1 (in trans). NT: no treatment; LP: losartan prenatal; AP: apocynin prenatal onset treatments. (PDF) [file pgen.1002458.s008.pdf]

Table S6: Relative mRNA levels of AngII/Ren pathway genes in different tissues

Heart

Ace

Genotype	Intervention	Mean	SD	P vs WT	P vs DD
WT	NT	1.25	0.24		
WT	LP	0.63	0.14	**0.001**	
WT	AP	0.67	0.36	**0.025**	
DD	NT	2.79	0.53	**0.000**	
DD	LP	0.82	0.19	0.145	**0.000**
DD	AP	0.90	0.48	1.000	**0.000**
DD/*Ncf1*-	NT	2.12	0.65	**0.028**	0.106
DD/*Ncf1*-	LP	0.54	0.12	1.000	**0.021**
DD/*Ncf1*-	AP	0.78	0.42	1.000	1.000

Agt

Genotype	Intervention	Mean	SD	P vs WT	P vs DD
WT	NT	1.59	0.21		
WT	LP	0.48	0.07	**0.000**	
WT	AP	0.76	0.25	**0.000**	
DD	NT	3.66	0.73	**0.000**	
DD	LP	0.42	0.05	0.398	**0.000**
DD	AP	1.53	0.50	**0.010**	**0.000**
DD/*Ncf1*-	NT	2.59	0.39	**0.007**	**0.002**
DD/*Ncf1*-	LP	0.39	0.07	0.066	1.000
DD/*Ncf1*-	AP	0.96	0.37	1.000	0.067

Ren

Genotype	Intervention	Mean	SD	P vs WT	P vs DD
WT	NT	0.70	0.24		
WT	LP	1.44	0.33	**0.003**	
WT	AP	1.16	0.22	**0.005**	
DD	NT	1.63	0.56	**0.002**	
DD	LP	2.89	0.73	**0.002**	**0.008**
DD	AP	2.38	0.53	**0.000**	0.158
DD/*Ncf1*-	NT	0.89	0.28	1.000	**0.014**
DD/*Ncf1*-	LP	1.61	0.64	1.000	**0.006**
DD/*Ncf1*-	AP	1.41	0.20	0.760	**0.001**

Table S6: Relative mRNA levels of AngII/Ren pathway genes in different tissues

Aorta

Ace

Genotype	Intervention	Mean	SD	P vs WT	P vs DD
WT	NT	1.52	0.31		
WT	LP	0.67	0.09	**0.000**	
WT	AP	0.91	0.06	**0.000**	
DD	NT	3.25	0.69	**0.001**	
DD	LP	0.80	0.28	0.616	**0.000**
DD	AP	1.29	0.23	**0.003**	**0.000**
DD/*Ncf1*-	NT	2.32	0.76	0.118	0.060
DD/*Ncf1*-	LP	0.54	0.08	0.604	0.053
DD/*Ncf1*-	AP	0.87	0.16	1.000	**0.001**

Agt

Genotype	Intervention	Mean	SD	P vs WT	P vs DD
WT	NT	0.64	0.24		
WT	LP	0.31	0.07	**0.034**	
WT	AP	0.73	0.29	1.000	
DD	NT	1.53	0.49	**0.001**	
DD	LP	0.37	0.08	0.660	**0.000**
DD	AP	1.47	0.56	**0.018**	1.000
DD/*Ncf1*-	NT	1.05	0.15	0.221	**0.028**
DD/*Ncf1*-	LP	0.34	0.08	1.000	1.000
DD/*Ncf1*-	AP	0.85	0.29	1.000	0.051

Ren

Genotype	Intervention	Mean	SD	P vs WT	P vs DD
WT	NT	1.28	0.28		
WT	LP	1.75	0.29	0.058	
WT	AP	1.39	0.29	1.000	
DD	NT	3.31	0.81	**0.000**	
DD	LP	3.40	0.71	**0.000**	1.000
DD	AP	2.57	0.52	**0.000**	0.248
DD/*Ncf1*-	NT	1.42	0.30	1.000	**0.000**
DD/*Ncf1*-	LP	2.03	0.34	1.000	**0.001**
DD/*Ncf1*-	AP	1.47	0.27	1.000	**0.000**

Table S6: Relative mRNA levels of AngII/Ren pathway genes in different tissues

Lung

Ace

Genotype	Intervention	Mean	SD	P vs WT	P vs DD
WT	NT	3.78	0.74		
WT	LP	2.63	0.14	**0.000**	
WT	AP	3.08	0.24	0.056	
DD	NT	8.66	1.14	**0.000**	
DD	LP	3.66	0.35	**0.000**	**0.000**
DD	AP	4.90	0.48	**0.000**	**0.000**
DD/*Ncf1*-	NT	6.44	1.19	**0.002**	**0.007**
DD/*Ncf1*-	LP	2.54	0.12	1.000	**0.000**
DD/*Ncf1*-	AP	4.67	0.51	**0.000**	1.000

Agt

Genotype	Intervention	Mean	SD	P vs WT	P vs DD
WT	NT	1.73	0.21		
WT	LP	0.62	0.14	**0.000**	
WT	AP	1.46	0.12	0.320	
DD	NT	3.90	1.09	**0.000**	
DD	LP	0.61	0.17	1.000	**0.000**
DD	AP	2.99	0.42	**0.001**	**0.037**
DD/*Ncf1*-	NT	2.93	0.17	**0.009**	**0.022**
DD/*Ncf1*-	LP	0.57	0.22	1.000	1.000
DD/*Ncf1*-	AP	2.16	0.82	0.115	0.054

Ren

Genotype	Intervention	Mean	SD	P vs WT	P vs DD
WT	NT	0.45	0.11		
WT	LP	0.24	0.07	**0.000**	
WT	AP	0.59	0.22	1.000	
DD	NT	1.14	0.26	**0.000**	
DD	LP	0.51	0.07	**0.000**	**0.006**
DD	AP	1.16	1.30	**0.000**	0.077
DD/*Ncf1*-	NT	0.62	0.24	0.576	**0.002**
DD/*Ncf1*-	LP	0.20	0.05	0.878	**0.000**
DD/*Ncf1*-	AP	0.67	0.67	1.000	**0.001**

Table S6: Relative mRNA levels of AngII/Ren pathway genes in different tissues

Kidney

Ren

Genotype	Intervention	Mean	SD	P vs WT	P vs DD
WT	NT	0.76	0.18		
DD	NT	3.27	0.33	**0.000**	
DD/*Ncf1-*	NT	0.74	0.05	0.802	**0.000**
