# Supplementary material for: Reduction of NADPH-Oxidase Activity Ameliorates the Cardiovascular Phenotype in a Mouse Model of Williams-Beuren Syndrome
Source: PLoS Genet. 2012 Feb 2;8(2):e1002458. doi: 10.1371/journal.pgen.1002458 (PMC3271062; doi:10.1371/journal.pgen.1002458)
Supplement: Table S7 — Quantification of protein nitrosylation and DHE fluorescence in the aortic wall. Mean and SD values of fluorescence intensity per area in the different groups of genotypes and interventions are shown, expressed as arbitrary units of fluorescence (Figure 2F and Figure 4A, 4B). Background effect was minimized by subtraction of the values obtained in negative control samples (no DHE). All aortic wall sections studied were prepared simultaneously and using identical experimental conditions to avoid experimental biases. Statistical analyses were done using ANOVA with a post hoc Bonferroni comparison among multiple groups. P-values of the different comparisons are also shown, with significant values displayed in bold. WT: wild-type; DD: distal deletion; DD/Ncf1−: double heterozygous for DD and Ncf1 (in trans); NT: no treatment; LN: losartan postnatal; LP: losartan prenatal; AN: apocynin postnatal; AP: apocynin prenatal. (PDF) [file pgen.1002458.s009.pdf]

Table S7: Quantification of protein nitrosylation and DHE fluorescence in aortic wall

Protein nitrosylation

Genotype	Intervention	Mean	SD	P vs WT-NT	P vs DD
WT	NT	16,384.05	3,378.25		
DD	NT	23,446.04	7,157.71	**0.000**	
DD/*Ncf1*-	NT	8,579.32	3,844.15	**0.000**	**0.000**

ROS production

Genotype	Intervention	Mean	SD	Relative Values	P vs WT	P vs DD
WT	NT	12,750.72	4,293.92	1.00		
WT	LP	9,116.88	6,026.33	0.72		
WT	LN	4,476.72	3,517.27	0.35		
WT	AP	6,186.58	3,498.34	0.49		
WT	AN	5,968.12	2,057.29	0.47		
DD	NT	17,043.07	5,574.25	1.34	**0.001**	
DD	LP	13,314.86	3,774.84	1.04	0.644	**0.017**
DD	LN	6,761.91	3,062.31	0.53	**0.000**	**0.000**
DD	AP	9,126.90	3,717.63	0.72	**0.010**	**0.000**
DD	AN	11,597.32	4,440.65	0.91	0.370	**0.000**
DD/*Ncf1*-	NT	6,688.86	2,768.13	0.52	**0.000**	**0.000**
DD/*Ncf1*-	LP	5,475.40	1,890.57	0.43	**0.000**	**0.000**
DD/*Ncf1*-	LN	2,777.75	1,369.01	0.22	**0.000**	**0.000**
DD/*Ncf1*-	AP	3,219.99	1,492.19	0.25	**0.000**	**0.000**
DD/*Ncf1*-	AN	3,252.79	1,119.66	0.26	**0.000**	**0.000**
