# Supplementary material for: Reduction of NADPH-Oxidase Activity Ameliorates the Cardiovascular Phenotype in a Mouse Model of Williams-Beuren Syndrome
Source: PLoS Genet. 2012 Feb 2;8(2):e1002458. doi: 10.1371/journal.pgen.1002458 (PMC3271062; doi:10.1371/journal.pgen.1002458)
Supplement: Table S8 — Relative mRNA levels of oxidative stress molecules in heart, aorta and lung. Relative mRNA levels (mean and SD values) of 5 NOX-related genes were recorded by qRT-PCR analysis in hearts, aortas and lungs (Figure 1B, 1C and Figure 4B) of 16-weeks-old mice. Each sample and the corresponding negative control for each pair of primers were analyzed in triplicate at least in two independent experiments. Statistical analyses with two-group comparisons were performed by t-test. P-values of the different comparisons are also shown, with significant values displayed in bold. DD: distal deletion (from Limk1 to Trim50); PD: proximal deletion (from Gtf2i to Limk1); D/P: double heterozygous for DD and PD deletions (in trans). NT: no treatment; LT: losartan treatment; AT: apocynin treatment. (PDF) [file pgen.1002458.s010.pdf]

Table S8: Relative mRNA levels of oxidative stress molecules in heart, aorta and lung

Heart

Ncf1

Genotype	Intervention	Mean	SD	P vs WT	P vs DD
DD	NT	2.74	0.37	**0.000**	
DD	LT	0.57	0.06	**0.000**	**0.000**
DD	AT	0.95	0.18	0.377	**0.002**
DD/*Ncf1-*	NT	1.10	0.03	1.000	**0.000**
PD	NT	0.70	0.05	0.346	**0.000**
D/P	NT	1.95	0.35	**0.000**	**0.017**

Ncf2

Genotype	Intervention	Mean	SD	P vs WT	P vs DD
DD	NT	1.92	0.20	**0.000**	
DD	LT	0.82	0.02	**0.000**	**0.000**
DD	AT	0.69	0.10	**0.000**	**0.000**
DD/*Ncf1-*	NT	0.73	0.10	**0.003**	**0.000**
PD	NT	1.10	0.13	0.737	**0.000**
D/P	NT	0.72	0.05	**0.000**	**0.000**

Nox2/Cybb

Genotype	Intervention	Mean	SD	P vs WT	P vs DD
DD	NT	1.99	0.19	**0.000**	
DD	LT	1.10	0.07	**0.004**	**0.030**
DD	AT	1.67	0.04	**0.000**	0.127
DD/*Ncf1-*	NT	1.28	0.07	0.232	**0.005**
PD	NT	1.13	0.09	1.000	**0.000**
D/P	NT	1.95	0.29	**0.000**	1.000

Nox4

Genotype	Intervention	Mean	SD	P vs WT	P vs DD
DD	NT	2.72	0.28	**0.002**	
DD	LT	0.91	0.07	**0.008**	**0.000**
DD	AT	2.15	0.21	**0.000**	0.062
DD/*Ncf1-*	NT	0.78	0.08	**1.000**	**0.027**
PD	NT	1.10	0.24	1.000	**0.028**
D/P	NT	1.35	0.76	1.000	0.060

Cyba

Genotype	Intervention	Mean	SD	P vs WT	P vs DD
DD	NT	1.15	0.14	1.000	
DD	LT	0.80	0.05	0.084	**0.015**
DD	AT	1.04	0.08	0.106	0.724
DD/*Ncf1-*	NT	0.64	0.02	0.280	0.102
PD	NT	1.12	0.27	1.000	1.000
D/P	NT	1.52	0.31	**0.002**	0.303

Rac2

Genotype	Intervention	Mean	SD	P vs WT	P vs DD
DD	NT	1.32	0.12	**0.000**	
DD	LT	0.96	0.10	0.246	**0.013**
DD	AT	1.27	0.07	**0.000**	1.000
DD/*Ncf1-*	NT	1.11	0.08	0.069	0.069

Table S8: Relative mRNA levels of oxidative stress molecules in heart, aorta and lung

Aorta

Ncf1

Genotype	Intervention	Mean	SD	P vs WT	P vs DD
DD	NT	2.34	0.18	**0.000**	
PD	NT	0.76	0.14	0.068	**0.000**
D/P	NT	1.62	0.13	**0.002**	**0.003**

Ncf2

Genotype	Intervention	Mean	SD	P vs WT	P vs DD
DD	NT	1.78	0.07	**0.002**	
PD	NT	0.86	0.14	0.100	**0.004**
D/P	NT	0.96	0.17	0.762	**0.026**

Nox2/Cybb

Genotype	Intervention	Mean	SD	P vs WT	P vs DD
DD	NT	1.81	0.03	**0.011**	
PD	NT	1.03	0.02	0.770	**0.000**
D/P	NT	1.94	0.12	**0.002**	0.258

Nox4

Genotype	Intervention	Mean	SD	P vs WT	P vs DD
DD	NT	2.52	0.02	**0.000**	
PD	NT	1.04	0.11	0.701	**0.001**
D/P	NT	1.62	0.21	**0.028**	**0.016**

Cyba

Genotype	Intervention	Mean	SD	P vs WT	P vs DD
DD	NT	1.03	0.10	0.233	
PD	NT	0.99	0.07	0.911	0.371
D/P	NT	1.33	0.08	**0.032**	**0.006**

Table S8: Relative mRNA levels of oxidative stress molecules in heart, aorta and lung

Lung

Ncf1

Genotype	Intervention	Mean	SD	P vs WT	P vs DD
DD	NT	2.71	0.23	**0.000**	
PD	NT	0.69	0.01	**0.010**	**0.000**
D/P	NT	1.75	0.34	**0.021**	**0.014**

Ncf2

Genotype	Intervention	Mean	SD	P vs WT	P vs DD
DD	NT	1.86	0.24	**0.018**	
PD	NT	1.02	0.11	0.824	**0.042**
D/P	NT	1.02	0.07	0.223	**0.016**

Nox2/Cybb

Genotype	Intervention	Mean	SD	P vs WT	P vs DD
DD	NT	2.40	0.14	**0.004**	
PD	NT	1.10	0.02	0.111	**0.004**
D/P	NT	2.07	0.36	**0.045**	0.285

Nox4

Genotype	Intervention	Mean	SD	P vs WT	P vs DD
DD	NT	2.21	0.29	**0.003**	
PD	NT	0.95	0.11	0.622	**0.012**
D/P	NT	1.63	0.15	0.094	0.149

Cyba

Genotype	Intervention	Mean	SD	P vs WT	P vs DD
DD	NT	1.58	0.13	**0.018**	
PD	NT	0.95	0.10	0.623	**0.005**
D/P	NT	0.99	0.02	0.923	**0.013**
