# Supplementary material for: Reduction of NADPH-Oxidase Activity Ameliorates the Cardiovascular Phenotype in a Mouse Model of Williams-Beuren Syndrome
Source: PLoS Genet. 2012 Feb 2;8(2):e1002458. doi: 10.1371/journal.pgen.1002458 (PMC3271062; doi:10.1371/journal.pgen.1002458)
Supplement: Table S9 — Secondary effects of pharmacological treatments. Prenatal outcomes and premature postnatal deaths in treated animals. We found a high proportion of fetal deaths (∼32%) only associated with the prenatal administration of losartan, calculated by the expected number of pups born by mate. No differences among genotypes were observed. (PDF) [file pgen.1002458.s011.pdf]

Table S9: Secondary effects of pharmacological treatments

Prenatal outcomes (birth rates) after pharmacological intervention

Intervention	Nº of crosses	Pups number	pups / cross
**NT**	16.00	57.00	3.56
**LP**	35.00	85.00	2.43
**AP**	24.00	79.00	3.29

Expected	Observed	%
124.69	85.00	68.17
85.50	79.00	92.40

Intervention	Mean	SD	*P*
**NT**	3.80	1.15	
**LP**	2.50	1.33	**0.00**
**AP**	3.43	1.44	0.41

NT: no treatment; LP: losartan prenatal; AP: apocynin prenatal

Premature postnatal deaths after prenatal treatment with losartan

Genotypes	Total Nº	Nº of Premature Death
WT	20	3
DD	20	4
DD/*Ncf1*-	14	2
