# Supplementary material for: Reduction of NADPH-Oxidase Activity Ameliorates the Cardiovascular Phenotype in a Mouse Model of Williams-Beuren Syndrome
Source: PLoS Genet. 2012 Feb 2;8(2):e1002458. doi: 10.1371/journal.pgen.1002458 (PMC3271062; doi:10.1371/journal.pgen.1002458)
Supplement: Table S10 — Primer sequences and PCR conditions for qRT-PCR and genotyping. The locus name, primer sequences, amplicon size, genomic location and the optimal melting temperatures for each specific primer are shown. Conditions for qRT-PCR are also shown, following the recommendations of the MIQE guidelines. (PDF) [file pgen.1002458.s012.pdf]

Table S10: Primer sequences and PCR conditions for qRT-PCR and genotyping

qRT-PCR

Gene name	Sequence (5'→3')	Amplicon size	Location	Tm
Rps28	TAGGGTAACCAAAGTGCTGGGC	104	exon 1-2	64.49
	GACATTTCGGATGATAGAGCGG		exon 3	63.02
Ace	GCCATCCTTCCTTTTTTCCCA	181	exon 16	64.45
	GCACATAGGCATGCAGGTTCA		exon17	64.38
Agt	TGTGACAGGGTGGAAGATGA	116	exon 1	60.09
	CAGGCAGCTGAGAGAAACCT		exon 2	59.75
Ren	CTCCTGGCAGATCACGATGAA	161	exon 6	63.62
	TTCTCTTCTCCTTGGCTCCCA		exon 7	63.54
Ncf1	TCCCTGCATCCTATCTGGAG	169	exon 7	60.17
	TCCAGGAGCTTATGAATGACC		exon 8	59.14
Ncf2	CCGACAAGAAGGACTGGAAG	219	exon 1	59.84
	CAAGGTCGTACTTCTCCATTCTG		exon 2-3	60.17
Nox2/Cybb	CATCGGTGACAATGAGAACG	229	exon 5	60.11
	AAGGCCGATGAAGAAGATCA		exon 6	59.77
Nox4	ATCTTTGCCTCGAGGGTTTT	182	exon 9	60.07
	TGACAGGTTTGTTGCTCCTG		exon 10	59.87
Cyba	AGATCGAGTGGGCCATGT	102	exon 1	59.00
	ACCACTGTGTGAAACGTCCA		exon2	60.00
Rac2	AACGCCTTCCCTGGAGAATA	150	exon 2	60.95
	TGTCTGTGGGTAGGAGAGTGG		exon 3	60.16
Eln	TCCCGGTGGAGTCTATTATCC	92	exon 2	60.16
	CTGGCTTAGGTGGTTTTCCTC		exon 4	60.12

**qRT-PCR Conditions**	Final volume 10 µl
Stage 1	50º-2 min
Stage 2	95º-10 min
Stage 3	(95º-30 sec; 58º-30 sec; 72º-30sec)X40
Stage 4	72º- 7 min
Dissociation stage	95º-15 sec; 60º-15sec; 95º-15sec

Genotyping

Locus	Sequence (5'→3')	Amplicon size
Ncf1	TGGAAGAAGCTGAGAGTTGAGG	160
	TCCAGGAGCTTATGAATGACC	
DD deletion	AGAAGGTCCACCAGCTCAGT	458
	TTTCTGTGGGGCAAAATGTA	
PD deletion	TTTCTGTGGGGCAAAATGTA	352
	AACAGCCTGCCAACTTCTTT	
