# Supplementary material for: Reduction of NADPH-Oxidase Activity Ameliorates the Cardiovascular Phenotype in a Mouse Model of Williams-Beuren Syndrome
Source: PLoS Genet. 2012 Feb 2;8(2):e1002458. doi: 10.1371/journal.pgen.1002458 (PMC3271062; doi:10.1371/journal.pgen.1002458)
Supplement: Table S11 — Recording of drinking volumes. We recorded daily drinking volumes of all untreated and treated mice. The table displays the mean daily volume drank per animal in ml. Up to 4 littermate animals were stocked per cage, regardless of their genotype. No significant differences between groups were observed. NT: no treatment; LN: losartan postnatal treatment; AN: apocynin postnatal treatment. (PDF) [file pgen.1002458.s013.pdf]

Table S11: Recording of drinking volumes

**Intervention**	**Mean**	**SD**	***P***
**NT**	3.97	0.31	
**LN**	4.15	0.07	0.20
**AN**	3.96	0.13	0.96
